# Supplementary material for: Can primary care research be conducted more efficiently using routinely reported practice-level data: a cluster randomised controlled trial conducted in England?
Source: BMJ Open. 2022 Jul 1;12(7):e061574. doi: 10.1136/bmjopen-2022-061574 (PMC9252201; doi:10.1136/bmjopen-2022-061574)
Supplement: Author's manuscript [file bmjopen-2022-061574.draft_revisions.pdf]

BMJ Open

BMJ Open is committed to open peer review. As part of this commitment we make the peer review history of every article we publish publicly available.

When an article is published we post the peer reviewers' comments and the authors' responses online. We also post the versions of the paper that were used during peer review. These are the versions that the peer review comments apply to.

The versions of the paper that follow are the versions that were submitted during the peer review process. They are not the versions of record or the final published versions. They should not be cited or distributed as the published version of this manuscript.

BMJ Open is an open access journal and the full, final, typeset and author-corrected version of record of the manuscript is available on our site with no access controls, subscription charges or pay-per-view fees (http://bmjopen.bmj.com).

If you have any questions on BMJ Open's open peer review process please email info.bmjopen@bmj.com

BMJ Open

A more efficient approach to randomised controlled trials in primary care using routinely collected practice-level data

Journal:	*BMJ Open*
Manuscript ID	bmjopen-2022-061574
Article Type:	Original research
Date Submitted by the Author:	01-Feb-2022

Complete List of Authors:	Blair, Peter; University of Bristol, Population Health Sciences<br>Ingram, Jenny; University of Bristol, Bristol Medical School<br>Clement, Clare; University of Bristol, Bristol Medical School<br>Young, Grace; University of Bristol, Bristol Medical School; Bristol Randomised Trials Collaboration, University of Bristol<br>Seume, Penny; University of Bristol, Centre for Academic Primary Care, Bristol Medical School: Population Health Sciences<br>Taylor, Jodi; University of Bristol, Bristol Medical School<br>Cabral, Christie; University of Bristol, Centre for Academic Primary Care<br>Lucas, Patricia; University of Bristol, School for Policy Studies<br>Beech, Elizabeth; NHS Improvement<br>Horwood, Jeremy; University of Bristol, NIHR ARC West<br>Dixon, Padraig; Oxford University<br>Gulliford, Martin; King's College London, UK<br>Francis, Nick; University of Southampton, School of Primary Care Population Sciences and Medical Education<br>Creavin, Sam T; University of Bristol, Bristol Medical School<br>Lane, Athene; University of Bristol, School of Social and Community Medicine<br>Bevan, Scott; University of Bristol, Centre for Academic Primary Care, Bristol Medical School: Population Health Sciences<br>Hay, Alastair; University of Bristol, Centre for Academic Primary Care

Keywords:	PRIMARY CARE, Respiratory infections < THORACIC MEDICINE, STATISTICS & RESEARCH METHODS

SCHOLARONE™
Manuscripts

I, the Submitting Author has the right to grant and does grant on behalf of all authors of the Work (as defined in the below author licence), an exclusive licence and/or a non-exclusive licence for contributions from authors who are: i) UK Crown employees; ii) where BMJ has agreed a CC-BY licence shall apply, and/or iii) in accordance with the terms applicable for US Federal Government officers or employees acting as part of their official duties; on a worldwide, perpetual, irrevocable, royalty-free basis to BMJ Publishing Group Ltd ("BMJ") its licensees and where the relevant Journal is co-owned by BMJ to the co-owners of the Journal, to publish the Work in this journal and any other BMJ products and to exploit all rights, as set out in our licence.

The Submitting Author accepts and understands that any supply made under these terms is made by BMJ to the Submitting Author unless you are acting as an employee on behalf of your employer or a postgraduate student of an affiliated institution which is paying any applicable article publishing charge ("APC") for Open Access articles. Where the Submitting Author wishes to make the Work available on an Open Access basis (and intends to pay the relevant APC), the terms of reuse of such Open Access shall be governed by a Creative Commons licence – details of these licences and which Creative Commons licence will apply to this Work are set out in our licence referred to above.

Other than as permitted in any relevant BMJ Author's Self Archiving Policies, I confirm this Work has not been accepted for publication elsewhere, is not being considered for publication elsewhere and does not duplicate material already published. I confirm all authors consent to publication of this Work and authorise the granting of this licence.

For peer review only

A more efficient approach to randomised controlled trials in primary care using routinely collected practice-level data

Peter S Blair[1], Jenny Ingram[1], Clare Clement[2], Grace Young[2], Penny Seume[3], Jodi Taylor[2], Christie Cabral[3], Patricia J Lucas[4], Elizabeth Beech[5], Jeremy Horwood[3], Padraig Dixon[8], Martin Gulliford[6], Nick A Francis[7], Sam Creavin[3], J Athene Lane[2], Scott Bevan[2] & Alastair D Hay[3]

Author Affiliations

[1] Centre for Academic Child Health, Bristol Medical School, Population Health Sciences, University of Bristol

[2] Bristol Trials Centre (Bristol Randomised Trial Collaboration), Bristol Medical School, University of Bristol

[3] Centre for Academic Primary Care, Bristol Medical School: Population Health Sciences, University of Bristol.

[4] School for Policy Studies, University of Bristol

[5] NHS England and NHS Improvement South West

[6] Kings College, University of London

[7] Primary Care Research Centre, School of Primary Care Population Sciences and Medical Education, University of Southampton

[8] Nuffield Department of Primary Care Health Sciences, University of Oxford

Corresponding author:

Prof Peter S Blair (Professor of Epidemiology and Statistics), Centre for Academic Child Health, University of Bristol.

Address: Level D, St Michael's Hospital, Southwell St, Bristol, BS2 8EG

Phone: 01173425145 Fax: 01173425154 E-mail: p.s.blair@bris.ac.uk

Word Count (Main text): 3662 words

Key Words: Respiratory Tract Infection, Antibiotic Prescribing, Primary Care, Efficient Design

For peer review only

Abstract

Objectives: Conducting randomised controlled trials (RCTs) in primary care is challenging; recruiting patients during time-limited or remote consultations can increase selection bias and physical access to patients' notes is costly and time-consuming. We investigated barriers and facilitators to running a more efficient design.

Design: An RCT using an efficient design to reduce antibiotic prescribing among children presenting with acute cough and a respiratory tract infection with a clinician-focused embedded intervention at practice-level, avoiding recruitment of individuals, and using routinely collected data for the co-primary outcomes.

Setting: Primary care

Participants: Baseline data from GP practices and interviews with individuals from Clinical Research Networks (CRNs) in England who helped recruit practices and Clinical Commission Groups (CCGs) who collected outcome data.

Intervention: The intervention included: i) explicit elicitation of parental concerns, ii) the results of a prognostic algorithm to identify children at low risk of hospitalisation and iii) provision of a printout for carers including safety netting advice.

Co-Primary outcomes: For 0-9 year olds - i) Dispensing data for amoxicillin and macrolide antibiotics and ii) hospital admission rate for RTI.

Results: We recruited 294 of the intended 310 practices (95%) representing 336,496 registered 0-9 year-olds (5% of all 0-9 year-old children). Included practices were slightly larger, had slightly lower baseline prescribing rates and were more deprived than the English average. Engagement with CCGs and their understanding of their role in this research was variable. Engagement with CRNs and installation of the intervention was straight-forward although the impact of updates to practice IT systems and lack of familiarity required extended support in some practices. Data on the co-primary outcomes was almost 100%.

Conclusions: The infrastructure for trials at the practice level using routinely collected data for primary outcomes is viable in England and should be promoted for primary care research where appropriate.

Trial registration: ISRCTN11405239

For peer review only

Article Summary

Strengths and limitations of this study

- Utilising routinely collected data in primary care can be more efficient and may reduce problems with missing data whilst removing the burden of patient recruitment and potential for selection bias.
- Using the Clinical Research Network across England to recruit practices with variable research experience provides the opportunity for larger trials and findings that are more generalisable.
- Embedding the intervention within practice systems makes the tool easier to use and exploits patient data already on the system.
- This approach would not be suitable for patient focussed trials that require individual consent or for those using patient-reported outcomes as the primary outcome.
- A light touch approach requires less data to be collected (including denominator data such as patients consulting for different conditions) so is only viable if suitable proxy markers can be found.

For peer review only

Introduction

Conducting randomised controlled trials (RCTs) in primary care is essential for the development of a robust evidence base to improve the treatment, health and well-being of patients. However, it is a difficult environment in which to conduct research. In England, general practices are already divided into those who are willing to conduct research and those who are not which challenges the external validity of any successful trial when rolling out an intervention.[1] Effective recruitment strategies generally require practitioner involvement[2] but this is difficult in the time allowed for consultations and can lead to the exclusion of patients for whom recruitment might be more challenging and therefore increase the risk of selection bias.[3] The time pressure can be compounded if the intervention is not fully integrated into the practice computer systems; a stand-alone tool takes time to open and close, may not draw upon information already collected within the system and may be less amenable to modification on a wider scale. In Primary Care research there are also difficulties in collecting patient outcomes. This is largely dependent on physical access to patient notes and is both costly and time-consuming.

Conducting trials at the practice level (removing the need for clinicians to recruit individual patients) and using routinely collected data for primary outcomes may potentially provide for a more efficient design when these considerations are important. The research infrastructure in England created by the National Institute for Health Research (NIHR) could make this possible.[4] Clinical Research Networks (CRNs) support health and care organisations to be research active[5] and can help recruit practices at a national level, including those serving diverse socioeconomic populations, improving the generalisability of findings.[6] Nationally collected routine data are also available from Clinical Commissioning Groups (CCGs)[7] and reliable data related to activity or financial transactions, such as the dispensing of medications and hospitalisation rates can be used for primary trial outcomes. Over 90% of the 7526 GP practices in England use either the *Egton Medical Information Systems* (*EMIS*) (56%) or *SystmOne* (34%) and both have the facility to integrate intervention algorithms and utilise patient data already on the system.[8] Simpler designed studies, which place fewer demands on clinicians and practices compared with other studies, may also encourage research-naïve practices to take part in research.

In the search for a more efficient design to conducting RCTs in Primary Care we use, as an example, experiences from our on-going trial, which aims to reduce antibiotic prescribing among children presenting with an upper respiratory tract infection and cough (The CHIldren's COugh or CHICO trial) including semi-structured interviews with members from CRNs and CCGs. Recruitment at patient-level was found to be a challenge in our feasibility study.[9,10] A change to practice-level design

For peer review only - http://bmjopen.bmj.com/site/about/guidelines.xhtml

incorporating routine data in the main trial[11] and integrating the intervention within electronic record systems puts us in a position to look at the barriers and facilitators to this approach.

Methodology

Respiratory tract infections (RTIs) in children are common and present major resource implications for primary care.[12,13] Unnecessary use of antibiotics is associated with the development and proliferation of antimicrobial resistance. In 2016 our 5-year NIHR-funded 'TARGET' programme developed a prognostic algorithm to identify children with acute cough and RTI at very low risk of hospitalisation within 30 days and unlikely to need antibiotics.[14] The intervention included: i) explicit elicitation of parental concerns, ii) the results of the prognostic algorithm accompanied by prescribing guidance and iii) provision of support for a no-antibiotic strategy through a printout for carers including safety netting advice.[15]

The subsequent feasibility study, recruiting at the patient-level, showed prescribing reductions in both arms of the trial but also exposed the differential recruitment of significantly healthier children in the control arm. In the qualitative interviews, clinicians reported preferential recruitment of less unwell children as these were quicker to manage and therefore easier to recruit.[10]

To negate differential recruitment, and conserve resources, an 'efficient design' was proposed for the full trial. The main changes in design were:

i) Recruiting practices (with the help of CRNs and CCGs) rather than individual patients.
ii) Using routinely collected data from CCGs and from national reporting systems for the primary outcome rather than directly from the practices.
iii) Integrating the intervention within electronic medical records (with a triggered pop-up) rather than a stand-alone web-based tool.
iv) A light-touch approach to collecting secondary data using practice champions (eliciting the help of a practice manager or someone familiar with practice systems) rather than accessing patient notes.

The CHICO RCT is an efficient, pragmatic open label, two-arm (intervention vs. usual care) trial with an embedded qualitative study, aimed at reducing antibiotic prescribing amongst children presenting with acute cough and RTI, with randomisation at the practice level, using routine antibiotic dispensing and hospitalisation data to assess effectiveness. The study population is children aged 0-9 years presenting with acute cough and RTI. Oral suspensions are more often given to this age group. The setting is consultations in primary care practices with prescribing clinicians in diverse regions across England. Recruitment is at the practice level, so consent is not required for

For peer review only

individual participants. Recruitment of practices is via CCGs and by using the Clinical Research Network (CRN) who support patients, the public and health and care organisations across England to participate in high-quality research. Research ethics approval was given by London-Camden and Kings Cross Research Ethics Committee (ref:18/LO/0345). This research is funded by the National Institute for Health Research (NIHR) Health Technology Assessment (HTA) programme (funder ref: 16/31/98).

Semi-structured interviews were conducted via online meeting software with 5 participants including 3 members of a CRN network and 2 from CCGs. Participants had been involved in the set-up of CHICO including recruiting practices and obtaining routine data from CCGs. The interviews explored participants views and experiences of setting up the trial, including the roles of CCGs and CRNs, barriers and facilitators to practice recruitment, providing data, and conducting efficient design trials in primary care. Interviews were audio-recorded, transcribed verbatim and analysed using a framework approach.[16] Transcripts were all read for familiarisation with the data and an initial sample of transcripts using both deductive (key issues in the interview topic guide), and inductive (derived from the data) codes. The initial codes were discussed with the wider team and a thematic framework was developed and used to code the whole of the dataset. Findings were then summarised in table format and used to inform wider understanding of the facilitators and barriers to conducting CHICO.

Patient and Public Involvement

This intervention has been developed collaboratively with our parent advisory group (PAG) and clinical advisory group (CAG) throughout the 'TARGET' programme. Their comments and suggestions about the format of the intervention and parent/carer materials have informed the intervention and the design of the earlier feasibility study and the design of the main trial.

Results

i) Recruiting practices

The sample size calculation indicated we needed 310 practices (155 intervention, 155 usual care). We recruited 294 practices (94.8%) representing 336,496 registered patients aged 0-9 years old (5.0% of all 0-9 year-old children in England).[17] Practices were recruited using all 15 CRNs in England and 47 of the 211 CCGs covering the English regions in 2019 (Figure 1).[18] Over half of the practices (59%) were larger than the average list size in England, around a third (32%) had higher prescribing rates than the national average and over a quarter of the practices (26%) were in the most deprived socio-economic quintile (Table 1).

For peer review only

Table 1. Practice baseline characteristics

	n	CHICO practices (n=294) n (%) or *Median (IQR)*
Total number of 0-9 year olds listed[a]		339,496
Practice characteristics, using routine data sources		
Above the average list size in England[b]	294	173 (59%)
Median list size of 0-9 year olds (IQR)[a]	294	*984 (678, 1405)*
Above the median prescribing rate in England[c]	294	93 (32%)
Median prescribing rate (IQR)[d]	294	*17.9 (14.3, 24.6)*
Patient characteristics, using a practice questionnaire [e]		
Median # of General Practitioners	290	*6.0 (4.0, 9.0)*
Median # of salaried nurses	238	*2.0 (1.0, 4.0)*
Median # of sessional nurses	133	*0.0 (0.0, 1.0)*
Median # pharmacist independent prescribers	190	*1.0 (0.0, 1.0)*
Median # of Locums in previous 12 months	203	*3.0 (2.0, 6.0)*
Median distance to the nearest childrens A&E (miles)	287	*4.5 (2.2, 10.0)*
IMD[f] quintile based on practice postcode		
1 (most deprived)		77 (26%)
2		60 (20%)
3	294	60 (20%)
4		54 (18%)
5 (least deprived)		43 (15%)
Income Deprivation Affecting Children Index Score[g]		
Median proportion (IQR)	294	*15% (8%, 26%)*

[a] Based on the mean practice list size of 0-9 year olds over the 12-months prior to randomisation, [b] The median practice list size of 0-9 year olds in England in October 2019, the mid-point of recruitment, was 852 patients[14] , [c] Based on amoxicillin and macrolide use in England CCGs (median=22) for all ages[28]. [d] Based on the total number of amoxicillin/macrolide items dispensed over the 12-months prior to randomisation, divided by the practice list size, [e] As reported by the practice champion, [f] index of multiple deprivation, [g] The Income Deprivation Affecting Children Index (IDACI) measures the proportion of all children aged 0-15 living in income deprived families. It is a subset of the Income Deprivation Domain which measures the proportion of the population in an area experiencing deprivation relating to low income. The definition of low income used includes both those that are out-of-work, and those that are in work but who have low earnings (and satisfy the respective means tests).

Assuming most families live close to their GP practice, using Income Deprivation Affecting Children Index (IDACI), we estimate 15% of children included in the trial lived in a deprived neighbourhood (defined as those people that are out-of-work, and those that are in work but who have low earnings). Typically, the number of registered 0-9 year olds was just under 1000 per practice, staffed with a median of 6 general practitioners, 2 salaried nurses, 1 pharmacist and 3 locums over one year. The practice list size of 0-9 year old children ranged from 149 to 6969 with 64 practices (22%) having more than 1500 children registered (Figure 2a). The number of amoxicillin or macrolides dispensed, over the 12-month baseline period prior to randomisation ranged from <5 to >55 per 100 patients (Figure 2b) with a median of 18 items (Table 1). Recruitment was planned over a 12-month period but took 24 months to complete. This in part was due to first having to obtain agreement from CCGs (providing the co-primary outcomes) to participate in the study and delays due to the COVID-19 pandemic.

Facilitators

The 15 CRNs were helpful with the recruitment of practices (Table 2); presenting our study at CRN meetings and using the national CRN lead for support helped facilitate a co-ordinated national approach.

For peer review only

Table 2. Semi-structured interviews with members of CRNS and CCG

Question	Response
What are the facilitators to practice recruitment?	*"Communicating with the practices. I think in an academic centre, if you're writing to practices, they don't really know you, do they? Whereas actually, if we put it in our local bulletins and keep encouraging practices to think about it, then it's probably a better way of increasing uptake."* [P4 CCG]<br><br>*"There's been a nice friendship [with study team] and trying to create the right words to share with practices."* [P1 CCG]<br><br>*"Just keep on plugging. I think quite often they're either going through a really busy time, I've contacted the wrong person or there's not enough information there for them to make a snap decision. The more information I've got and the simpler I make it, then the more likely they are to say yes or no."* [P3 CRN]
What are the barriers to practice recruitment?	*"To be honest it was a little bit more difficult to get the paperwork signed by the CCG as well within the practices, 'cause a lot of the practices we generally have a named contact, someone who already knows us and we've got relationships with…so it was a lot easier to get engagement than it was from CCGs."* [P2 CRN]<br><br>*"I try to avoid the practice managers making the decisions if I can because they are good gatekeepers. Well our practice is very busy, that GP won't be interested when actually sometimes they quite often are."* [P3 CRN]
Tell us about Research active and non-active (naïve) practices?	*"It's usually the research active sites that get back to us but sometimes others do respond and want to take part in studies and we've got this ongoing engagement programme with all the practices in ((city)) to try and get more of them on board with research even if it's just doing simple stuff."* [P5 CRN]<br><br>*"I would say that about a third of the practices were what I would call research naïve or inexperienced, green as in they hadn't really had a sort of established relationship with us in the past."* [P2 CRN]
Tell us about the role of CCGs in Research?	*"I mean in reality they [CCG] don't play a major part in – and never actually have a major role in research. It's not a core business of a CCG like it would be in a provider. So we don't have a research department, which is probably why the CHICO trial ended up at my door because it was about prescribing."* [P4 CCG]
Tell us about efficient design trials?	*"Yeah, go for it because once you've got the practice on board, it's almost like they don't have to do as much either, I would definitely encourage practices to take on this type of research, definitely, as long as you've got everybody on board and it all works through then yeah, there's no reason why not."* [P3 CRN]
	"It seems quite suited to primary care because I think primary care's biggest issue is time. So, if they've got a patient in front of them, the chance of them actually getting that patient to consent is probably quite low because it's just they – that's an extra minute on a ten-minute appointment isn't it really?" [P4 CCG]

Some CRNs contact practices that opt-in to conduct research whilst others contact all the practices in their area (Table 2). Feedback from 11 of the 15 CRNs suggested around half the practices had joined the Research Sites Initiative scheme (NIHR funded scheme to enable research delivery) and were often the first to be contacted. Recruitment via CCGs had a wider reach of practices than via

For peer review only

CRNs, although CCG participation in recruitment was more variable depending on capacity. Using quarterly study newsletters for practices, CRNs and CCGs with league tables monitoring levels of recruitment improved responses.

Barriers

Limiting the contact to practices that want to opt-in to research via some of the CRNs misses the opportunity of letting research-naïve practices know about light-touch efficient design studies. The level of engagement from CRNs varied slightly but much more between CCGs. Some CCGs were averse to getting involved in research or cited lack of capacity as a reason to be excluded from the research (Table 2). It was difficult to know which individuals to contact and how to do so (some CCGs did not appear to be public-facing), response times were often slow, their role in research was often misunderstood, staff changes hindered communication and a number of CCGs merged during the study period. At the start of recruitment, October 2018, there were 211 CCGs across England. By the end of follow up, September 2021, there were only 106 CCGs. While this did reduce the number of CCG contacts required to obtain the data, it sometimes resulted in change of staff who were not familiar with the trial or its requirements. Of the 294 practices we recruited, we are aware of at least 22 practices (7%) who merged during their baseline/follow up annual data capture. We excluded practices who anticipated a merger with another practice but had no control over this once the practice was randomised, especially in the rare instances when the merging practices were randomised to different arms of the trial. The length of time from expression of interest from practices to randomisation was longer than expected due to the delayed site agreements returned from the practices.

<u>ii) Using routinely collected data for the primary outcomes</u>

The co-primary outcomes in the trial were i) practice dispensed prescription data for amoxicillin and macrolide antibiotics for children 0-9 years and ii) hospital admission rate for RTI among 0-9 year olds. The antibiotic dispensing rates were retrieved from the *NHSBSA ePACT2* reporting system for all practices in the trial and practice list size data by 5 year epoch was retrieved from *NHS Digital* and combined to create a dispensed prescription rate for amoxicillin and for macrolide antibiotics.[19] The hospital admission rates are routinely collected by all English CCGs. The hospital admission rates are routinely collected by all English CCGs. The ascertainment of these data was 99.7% (293/294) for antibiotic dispensing and hospitalisation rates. Data for one practice was only lost due to a merger.

Facilitators

Using aggregated data avoids the need for the consulting physician to solicit individual consent, reduces or eliminates the risk of selection bias and removes the task of accessing individual patient

For peer review only - http://bmjopen.bmj.com/site/about/guidelines.xhtml

notes. Routine data are often collected monthly so data completeness and quality can be monitored throughout the collection period, which was particularly important in this trial to scrutinise and report any sudden changes in hospital admission rates to the Data Monitoring Committee. The routinely collected data can be imported to data management software, thus there is less likelihood of data entry errors and missing data are rare. The cost of research staff and time taken to collect the primary outcome data was much reduced compared to a traditional trial. In total 11/294 practices asked to be withdrawn from the study (3.7%) and 14/294 (4.8%) were lost to follow-up from the study; the main reasons cited being lack of capacity and prioritising the COVID-19 pandemic. A further advantage of collecting routine data is that withdrawal of a practice does not necessarily mean a loss of the primary outcome data for that practice (if the data are already in the public domain).

Barriers

A potential problem with aggregate data collected from a third party is where data are suppressed, owing to a low number of events, although in this study data were collected over a 12-month period largely avoiding this problem. Some liaison was needed between the trial team and the CCGs to know exactly what data were available and in what format this could be presented. Dispensed prescription data, practice list size data and hospital admission data are reliable as they feed into financial transactions, however data reporting emergency department [ED] attendance were less so due to a limited coding set. Many ED attendances do not have a diagnosis coded, therefore the number of attendances attributable to RTI is likely to be inaccurate.

<u>iii) Integrating the intervention within the electronic medical record system</u>

The trial only included practices using the *EMIS* system. The practice champion was given written instructions on how to install the intervention within *EMIS*. Self-directed training materials were provided for all clinicians using the intervention. The algorithm consists of seven predictors of future hospitalisation, two of which were already in the practice systems (child age and history of asthma) and thus pulled into the algorithm automatically from patient records, and five which were entered during consultation (illness duration, raised temperature, vomiting in last 24 hours, presence of wheeze and presence of intercostal or subcostal recession).[20] Carer concerns were elicited during the consultation and formed part of a personalised leaflet generated from the system containing an easy to read 'Caring for Children with Coughs' graphic and safety-netting advice about when to seek medical care or advice.[16] Intervention use was monitored using searches on the *EMIS* system, run by the practice champion and shared with the research team.

Facilitators

For peer review only

Installation of the intervention was relatively straight-forward for *EMIS*. Practice systems allow for user-friendly manipulation so interventions can be integrated and interface with system data already collected, thus negating any additional clinical workload. Screen pop-ups can also be used to notify clinicians of eligible patients to the study.

Barriers

Familiarity with the *EMIS* system varied between practices thus the level of support required from the research team to help install the intervention and download usage also varied. Provision to help install or use third-party algorithms is not offered by system providers. EMIS upgrades to the system during the trial meant that rewriting installation instructions, resources and testing had to be carried out and fed back to the practices. For instance, the READ codes used to identify clinical terms within consultations were upgraded by EMIS in the early months of 2020 which meant the algorithm had to be amended so the intervention would function correctly. We found that our funded provision of IT support throughout the study was crucial to the smooth running of the integrated intervention. Although pop-ups can be used, some clinicians found them irritating and switched them off whilst other practices had so many pop-ups (especially during the COVID-19 pandemic) that the CHICO pop-up was often obscured.

<u>iv) A light-touch approach to data collection</u>

Clinicians were not required to provide any data about individual participants (apart from reporting serious adverse events), those in the intervention group were asked to familiarise themselves with the tool and use it, whilst those in the control arm were asked to provide usual care. Data collection from the practices directly was limited to short baseline and follow-up questionnaires. Data on intervention usage were downloaded from *EMIS* and the co-primary outcome downloads from ePACT2 and the relevant CCGs.

Facilitators

The light touch approach reduced the time needed during consultation to record information, the trawling of patient notes and provided a more objective data resource downloaded from the system rather than from individual input. Practice champions, familiar with practice systems, played an important role in obtaining the required data. Baseline questionnaires were received from 294/294 practices (100%), follow-up questionnaires were received from 265/294 practices (90%) whilst intervention usage was collected from 116/144 of the intervention practices (81%), indicating the data burden was not too onerous.

For peer review only

Barriers

Conversely, this approach reduced the level of interpretation that can be gleaned from the data. Removing patient level recruitment results in a loss of denominator data, in our case not knowing how many individual children consulted for RTI and cough. As a proxy we used the number of children registered at each practice. Given the diversity of the practices included in the trial we are assuming that those children taking part in the trial were no different from children in the general population, but we have no way of testing this assumption. We also lost detail that may be taken from the consultation of whether antibiotics were prescribed immediately or delayed relying instead on the number of antibiotics being dispensed. The lack of contact with usual care practices (apart from reminders to provide SAE reports) runs the risk of practices wanting to withdraw from the study, especially with changes of staff. Consideration also needs to be given to the ethical implications of not seeking consent from individual patients.

Discussion

The National Institute for Health Research (NIHR) is keen to see the design, development and delivery of more efficient, faster, innovative studies which provide robust evidence to inform clinical practice and policy.[21] The CHICO study demonstrates that an efficiently designed practice-level large trial in primary care using routinely collected data is feasible and potentially good value for money. The average cost of an HTA RCT was £1.25 million in 2019/20,[22] whereas the cost of the CHICO RCT, which included over 300,000 children (5% of the entire national 0-9 year-old population) and 4% of all GP practices in England, was below £1 million. Utilising routinely collected data as the primary outcome reduces problems with missing data whilst removing the burden of patient recruitment and focusing the clinician's time on using the intervention reflects real life practice.

Integrating the intervention within the practice system both exploits the data already available and adds to the patient's record avoiding duplicating of effort and saves time. Primary care practices are often very busy and the average length of face-to-face consultation in the UK is around 10 minutes; less than half the time given to patients in, for example, Sweden and the US.[23] Growing demands on primary care services have also led to policymakers promoting telephone and video consultations, even before the COVID-19 pandemic, and these sometimes don't lend themselves to enlisting patients in research.[24] Reducing the research burden for participants and clinicians is always desirable, but particularly so given the increasing time constraints in primary care. This light-touch approach may also be more appealing for practices who maybe research naïve; investing in different

For peer review only

recruitment strategies[25] using existing networks could potentially yield a more representative sample than previous trials in primary care from which to generalise any findings.

Efficient design studies which utilise routine data and recruit at practice level face their own challenges. First, this approach would not be suitable if individual patient consent is required or for trials using a patient reported outcome as the primary outcome, as these are not widely collected in routine care. Furthermore, the number of CCGs in England almost halved during the study period due to organisational restructuring which made it difficult to administer the trial. From March 2022 Integrated Care Systems will replace CCGs and this will provide new bureaucracy for researchers to develop relationships with. There is a lack of uniformity when approaching these commissioning groups and their role in research needs clarifying.[26] CRNs are more research-focused and can help with recruitment although adoption of the study needs to be made more explicit and a consistent national approach to include research naïve practices needs to be adopted within this network.

If practices are being used as the unit of analysis, then the commitment to research they have signed up for needs to be strengthened. Around 2.5% practices close or merge each year in England whilst some new practices open and recognition of the current research portfolio needs to be part of this process.[27] We were also surprised by the wide variability in practice list size, 8% of practices in the study having 3 sites or more. This has implications for future trials in terms of factoring in variable list sizes for sample size calculations and checking that multiple sites use the same electronic record systems. A light touch approach is only viable if suitable proxy markers can be found. Losing denominator data such as patients consulting for different conditions will depend on the hypothesis being tested and needs to strike a balance between accuracy of what you are trying to measure and whether a proxy marker will deliver the population impact of the intervention.

If one of the main intentions of primary care research is to provide the clinician with better tools, then we need to work more closely with those who supply the toolbox (IT system providers) and the third parties who provide the data within which practices work. The infrastructure to conduct efficiently designed trials that do not require patient-reported outcomes in England is potentially viable but does require more investment in time and effort to make recruitment of practices and data collection more accessible to researchers.

For peer review only

Authors Contributions

AH, PB, PL, NF and JI were responsible for developing the research questions. PB, AH, JI, PL, CCab, CClem, EB, MG, JH, SC, AL and NF and are responsible for the study design and collection of data. PS and SB are responsible for study management and coordination. GY, PD and CClem are responsible for the analysis of the data. PB drafted the paper. All authors read, commented on and approved the final manuscript.

Acknowledgements

This study was designed and delivered in collaboration with the Bristol Randomised Trials Collaboration (BRTC), part of the Bristol Trials Centre, is in receipt of National Institute for Health Research CTU support funding. The University of Bristol is acting as the sponsor for this trial and the trial is hosted by the NHS Bristol, North Somerset and South Gloucestershire Clinical Commissioning Group (CCG). The authors would like to thank all General practices, CCGs and CRNs for their involvement in CHICO. The authors would also like to thank the members of the TSC and DMC.

Funding

This research is funded by the National Institute for Health Research (NIHR) Health Technology Assessment (HTA) programme (funder ref: 16/31/98). The views expressed are those of the authors and not necessarily those of the NIHR or the Department of Health and Social Care.

Competing interest

None declared

Data Sharing

No additional data available

Ethics Approval

Research ethics approval was given by London-Camden and Kings Cross Research Ethics Committee (ref:18/LO/0345).

For peer review only

References

1) Allcott H. Site Selection Bias in Program Evaluation. *The Quarterly Journal of Economics* August 2015;130 (3): 1117–1165.
2) Ngune I, Jiwa M, Dadich A, Lotriet J, Sriram D. Effective recruitment strategies in primary care research: a systematic review. *Qual Prim Care* 2012;20(2):115-23
3) Foster JM, Sawyer SM, Smith L, Reddel HK, Usherwood T. Barriers and facilitators to patient recruitment to a cluster randomized controlled trial in primary care: lessons for future trials. *BMC Med Res Methodol* 2015 Mar 12;15:18
4) National Institute for Health Research https://www.nihr.ac.uk/researchers/collaborations-services-and-support-for-your-research/
5) Clinical Research Network https://www.nihr.ac.uk/explore-nihr/support/clinical-research-network.htm
6) Bower P, Grigoroglou C, Anselmi L, Kontopantelis E, Sutton M, Ashworth M, Evans P, Lock S, Smye S, Abel K. Is health research undertaken where the burden of disease is greatest? Observational study of geographical inequalities in recruitment to research in England 2013-2018. *BMC Med* 2020 May 18;18(1):133.
7) Clinical Commissioning Groups https://www.england.nhs.uk/ccgs/
8) Kontopantelis E, Stevens RJ, Helms PJ, Edwards D, Doran T, Ashcroft DM. Spatial Distribution of Clinical Computer Systems in Primary Care in England in 2016 and Implications for Primary Care Electronic Medical Record Databases: A Cross-Sectional Population Study. *BMJ Open* 2018 Feb 28;8(2):e020738
9) Turnbull SL, Redmond NM, Lucas P, Cabral C, Ingram J, Hollinghurst S, Hay AD, Peters TJ, Horwood J, Little P, Francis N, Blair PS. The CHICO (Children's Cough) Trial protocol: a feasibility randomised controlled trial investigating the clinical and cost-effectiveness of a complex intervention to improve the management of children presenting to primary care with acute respiratory tract infection. *BMJ Open* 2015 Sep 15;5(9):e008615.
10) Blair PS, Turnbull S, Ingram J, Redmond N, Lucas PJ, Cabral C, Hollinghurst S, Dixon P, Peters T, Horwood J, Little P, Francis NA, Gilbertson A, Jameson C, Hay AD. Feasibility cluster randomised controlled trial of a within-consultation intervention to reduce antibiotic prescribing for children presenting to primary care with acute respiratory tract infection and cough. *BMJ Open* 2017 May 9;7(5): e014506.
11) Seume P, Bevan S, Young G, Ingram J, Clement C, Cabral C, Lucas PJ, Beech E, Taylor J, Horwood J, Dixon P, Gulliford M, Francis NA, Creavin S, Lane AJ, Hay A, Blair PS. Protocol for an 'efficient design' cluster randomised controlled trial to evaluate a complex intervention to improve antibiotic prescribing for CHIldren presenting to primary care with acute COugh and respiratory tract infection: The CHICO study. *BMJ Open.* 2021 Mar 29;11(3): e041769.
12) Smith DRM, Dolk FCK, Pouwels KB, Christie M, Robotham JV, Smieszek T. Defining the appropriateness and inappropriateness of antibiotic prescribing in primary care. *J Antimicrob Chemother* 2018 Feb 1;73(suppl_2): ii11-ii18.
13) Vodicka TA, Thompson M, Lucas P, Heneghan C, Blair PS, Buckley DI, Redmond N and Hay AD. Reducing antibiotic prescribing for children with respiratory tract infections in primary care: a systematic review. *Br J Gen Pract* 2013 Jul;63(612): e445-54.
14) Hay AD, Redmond NM, Turnbull S, Christensen H, Thornton H, Little P, Thompson M, Delaney B, Lovering AM, Muir P, Leeming JP, Vipond B, Stuart B, Peters TJ, Blair PS. Development and internal validation of a clinical rule to improve antibiotic use in children

For peer review only

presenting to primary care with acute respiratory tract infection and cough: a prognostic cohort study. *Lancet Respir Med* 2016 Nov;4(11):902-910.

15) Lucas PJ, Ingram J, Redmond NM, Cabral C, Turnbull SL, Hay AD. Development of an intervention to reduce antibiotic use for childhood coughs in UK primary care using critical synthesis of multi-method research. *BMC Med Res Methodol*. 2017 Dec 28;17(1):175.
16) Ritchie J, Spencer L. Qualitative data analysis for applied policy research. In: A. Bryman and R. G. Burgess, ed. Analyzing qualitative data1994:173-94.
17) Population estimate of 0-9 year olds in England using October 2019 (mid-point of recruitment) https://digital.nhs.uk/data-and-information/publications/statistical/patients-registered-at-a-gp-practice/october-2019
18) CCG annual assessment 2019/20 © NHS England and NHS Improvement 2020 Publication approval reference: PAR112 https://www.england.nhs.uk/wp-content/uploads/2020/11/ccg-annual-assessment-report-19-20.pdf
19) ePACT2 prescribing data held by NHS Prescription Services. https://www.nhsbsa.nhs.uk/epact2
20) Hay AD, Redmond NM, Turnbull S, Christensen H, Thornton H, Little P, Thompson M, Delaney B, Lovering AM, Muir P, Leeming JP, Vipond B, Stuart B, Peters TJ, Blair PS. Development and internal validation of a clinical rule to improve antibiotic use in children presenting to primary care with acute respiratory tract infection and cough: a prognostic cohort study. *Lancet Respir Med*. 2016 Nov;4(11):902-910.
21) NIHR: Annual Efficient Studies funding calls for CTU projects. https://www.nihr.ac.uk/documents/ad-hoc-funding-calls-for-ctu-projects/20141
22) NIHR Annual Report 2019/20. Department of Health & Social Care (page 5) https://www.nihr.ac.uk/documents/about-us/our-contribution-to-research/research-performance/NIHR_Annual_Report_19_20.pdf
23) Irving G, Neves AL, Dambha-Miller H, Oishi A, Tagashira H, Verho A, Holden J. International variations in primary care physician consultation time: a systematic review of 67 countries. *BMJ Open* 2017 Nov 8;7(10):e017902
24) Hammersley V, Donaghy E, Parker R, McNeilly H, Atherton H, Bikker A, Campbell J, McKinstry B. Comparing the content and quality of video, telephone, and face-to-face consultations: a non-randomised, quasi-experimental, exploratory study in UK primary care. *Br J Gen Pract* 2019 Aug 29;69(686):e595-e604
25) Ellis SD, Bertoni AG, Bonds DE, Randall Clinch C, Balasubramanyam A, Blackwell C, Chen H, Lischke M, Goff Jr DC. Value of recruitment strategies used in a primary care practice-based trial. *Contemp Clin Trials* 2007 May;28(3):258-67.
26) Checkland K, McDermott I, Coleman A, Perkins N. Complexity in the new NHS: longitudinal case studies of CCGs in England. *BMJ Open* 2016 Jan 7;6(1):e010199
27) Practices closing or merging https://digital.nhs.uk/data-and-information/find-data-and-publications/supplementary-information/2018-supplementary-information-files/number-of-gp-practices-closed-or-merged-in-england-between-1-july-2016-and-31-december-2017-by-nhs-geographies
28) Estimate of antibiotic items prescribed per CCG (October 2017 to September 2018) https://openprescribing.net/analyse/#org=CCG&orgIds=15E,15C,D2P2L,14Y,06H,04Y,27D,15N,01A,05D,97R,D4U1Y,11M,D9Y0V,06N,91Q,11N,15F,99A,14L,01K,13T,06T,05G,W2U3Z,52R,10Q,01G,05Q,11X,M2L0M,05W,72Q,36L,05V,00P,92A,03Q&numIds=0501013B0,5.1.5&denom=total_list_size&selectedTab=summary

Figure 1. The CCGs taking part in the CHICO study (bold), shaded according to the number of items of amoxicillin and macrolides, per 1000 list size at the mid point of recruitment (Oct ’19).

Figure 2a. The distribution of practice list sizes*, of 0-9 year olds, for those practices taking part in the CHICO study

Figure 2b. The distribution of practice dispensing rates*at baseline, for 0-9 year olds, for those practices taking part in the CHICO study

For peer review only

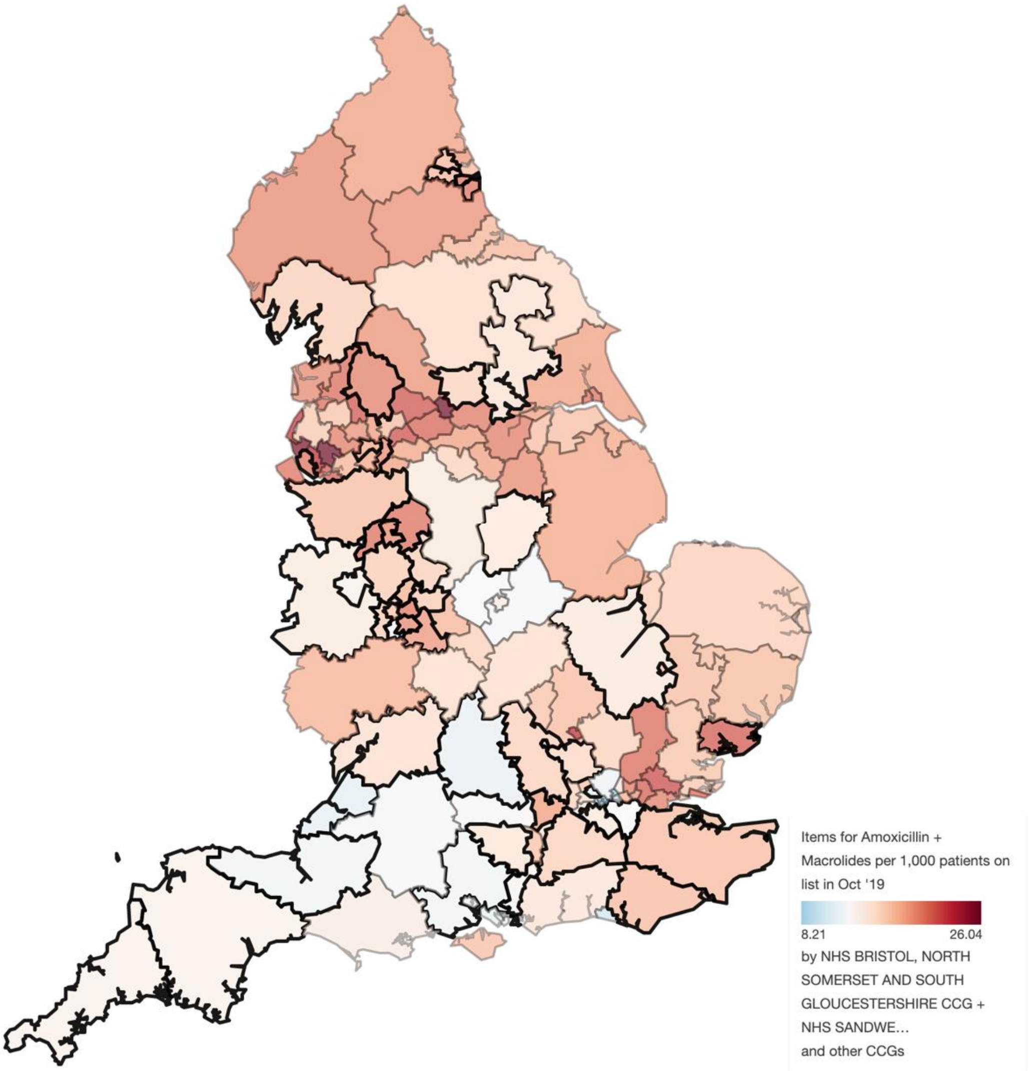

Source: OpenPrescribing.net, EBM DataLab, University of Oxford, 2017

For up to date data please refer to:
[https://openprescribing.net/analyse/#org=CCG&orgIds=15E,15C,D2P2L,14Y,06H,04Y,27D,15N,01A,05D,97R,D4U1Y,11M,D9Y0V,06N,91Q,11N,15F,99A,14L,01K,13T,06T,05G,W2U3Z,52R,10Q,01G,05Q,11X,M2L0M,05W,72Q,36L,05V,00P,92A,03Q&numIds=0501013B0,5.1.5&denom=total_list_size&selectedTab=map]

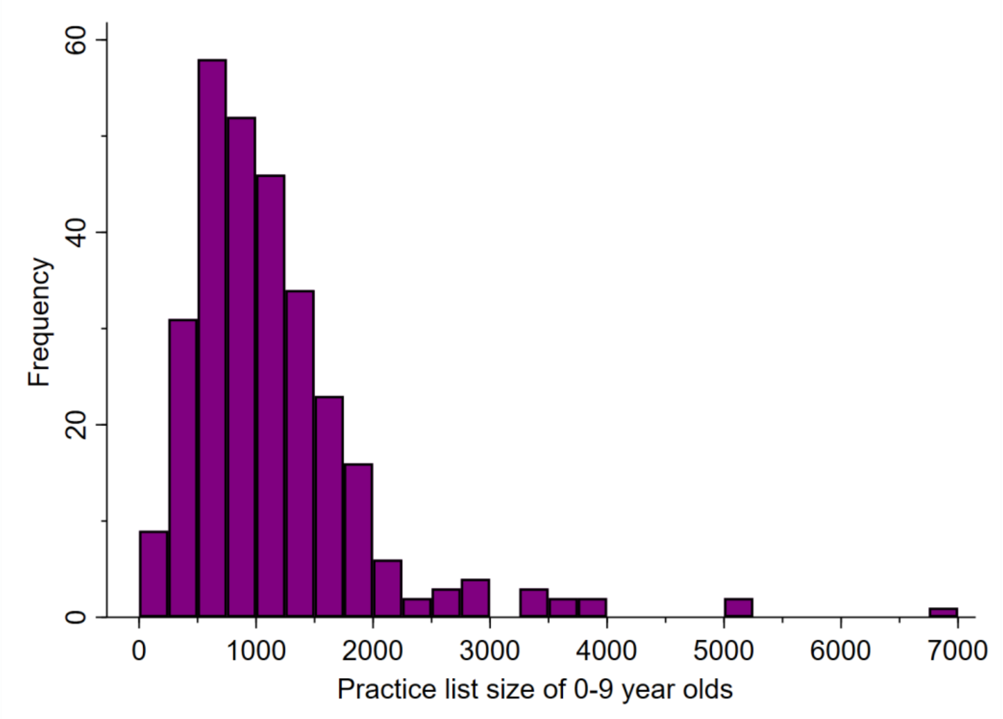

*Defined for each practice as the mean list size of 0-9 year olds, over the 12 months prior to randomisation (source: https://digital.nhs.uk/data-and-information/publications/statistical/patients-registered-at-a-gp-practice)

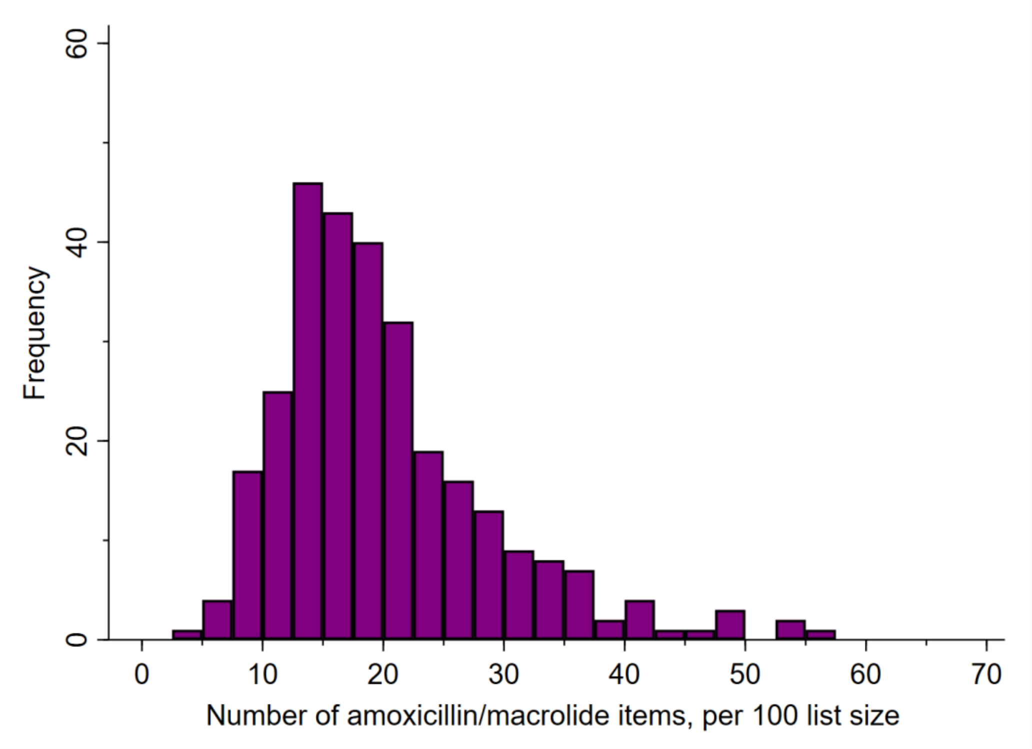

*Defined for each practice as the number of amoxicillin or macrolides dispensed, over the 12-month baseline period prior to randomisation, divided by the practice list size. This may include multiple items per child.

BMJ Open

Can randomised controlled trials be conducted more efficiently in primary care using routinely reported practice-level data?

Journal:	*BMJ Open*
Manuscript ID	bmjopen-2022-061574.R1
Article Type:	Original research
Date Submitted by the Author:	05-May-2022
Complete List of Authors:	Blair, Peter; University of Bristol, Population Health Sciences<br>Ingram, Jenny; University of Bristol, Bristol Medical School<br>Clement, Clare; University of Bristol, Bristol Medical School<br>Young, Grace; University of Bristol, Bristol Medical School; Bristol Randomised Trials Collaboration, University of Bristol<br>Seume, Penny; University of Bristol, Centre for Academic Primary Care, Bristol Medical School: Population Health Sciences<br>Taylor, Jodi; University of Bristol, Bristol Medical School<br>Cabral, Christie; University of Bristol, Centre for Academic Primary Care<br>Lucas, Patricia; University of Bristol, School for Policy Studies<br>Beech, Elizabeth; NHS Improvement<br>Horwood, Jeremy; University of Bristol, NIHR ARC West<br>Dixon, Padraig; Oxford University<br>Gulliford, Martin; King's College London, UK<br>Francis, Nick; University of Southampton, School of Primary Care Population Sciences and Medical Education<br>Creavin, Sam T; University of Bristol, Bristol Medical School<br>Lane, Athene; University of Bristol, School of Social and Community Medicine<br>Bevan, Scott; University of Bristol, Centre for Academic Primary Care, Bristol Medical School: Population Health Sciences<br>Hay, Alastair; University of Bristol, Centre for Academic Primary Care
**Primary Subject Heading**:	General practice / Family practice
Secondary Subject Heading:	Respiratory medicine
Keywords:	PRIMARY CARE, Respiratory infections < THORACIC MEDICINE, QUALITATIVE RESEARCH

SCHOLARONE™
Manuscripts

I, the Submitting Author has the right to grant and does grant on behalf of all authors of the Work (as defined in the below author licence), an exclusive licence and/or a non-exclusive licence for contributions from authors who are: i) UK Crown employees; ii) where BMJ has agreed a CC-BY licence shall apply, and/or iii) in accordance with the terms applicable for US Federal Government officers or employees acting as part of their official duties; on a worldwide, perpetual, irrevocable, royalty-free basis to BMJ Publishing Group Ltd ("BMJ") its licensees and where the relevant Journal is co-owned by BMJ to the co-owners of the Journal, to publish the Work in this journal and any other BMJ products and to exploit all rights, as set out in our licence.

The Submitting Author accepts and understands that any supply made under these terms is made by BMJ to the Submitting Author unless you are acting as an employee on behalf of your employer or a postgraduate student of an affiliated institution which is paying any applicable article publishing charge ("APC") for Open Access articles. Where the Submitting Author wishes to make the Work available on an Open Access basis (and intends to pay the relevant APC), the terms of reuse of such Open Access shall be governed by a Creative Commons licence – details of these licences and which Creative Commons licence will apply to this Work are set out in our licence referred to above.

Other than as permitted in any relevant BMJ Author's Self Archiving Policies, I confirm this Work has not been accepted for publication elsewhere, is not being considered for publication elsewhere and does not duplicate material already published. I confirm all authors consent to publication of this Work and authorise the granting of this licence.

For peer review only

Can randomised controlled trials be conducted more efficiently in primary care using routinely reported practice-level data?

Peter S Blair[1], Jenny Ingram[1], Clare Clement[2], Grace Young[2], Penny Seume[3], Jodi Taylor[2], Christie Cabral[3], Patricia J Lucas[4], Elizabeth Beech[5], Jeremy Horwood[3], Padraig Dixon[8], Martin Gulliford[6], Nick A Francis[7], Sam Creavin[3], J Athene Lane[2], Scott Bevan[2] & Alastair D Hay[3]

Author Affiliations

[1] Centre for Academic Child Health, Bristol Medical School, Population Health Sciences, University of Bristol

[2] Bristol Trials Centre (Bristol Randomised Trial Collaboration), Bristol Medical School, University of Bristol

[3] Centre for Academic Primary Care, Bristol Medical School: Population Health Sciences, University of Bristol.

[4] School for Policy Studies, University of Bristol

[5] NHS England and NHS Improvement South West

[6] Kings College, University of London

[7] Primary Care Research Centre, School of Primary Care Population Sciences and Medical Education, University of Southampton

[8] Nuffield Department of Primary Care Health Sciences, University of Oxford

Corresponding author:

Prof Peter S Blair (Professor of Epidemiology and Statistics), Centre for Academic Child Health, University of Bristol.

Address: Level D, St Michael's Hospital, Southwell St, Bristol, BS2 8EG

Phone: 01173425145 Fax: 01173425154   E-mail: p.s.blair@bris.ac.uk

Word Count (Main text): 3854 words (excluding tables)

Key Words: Respiratory Tract Infection, Antibiotic Prescribing, Primary Care, Efficient Design

For peer review only

Abstract

Objectives: Conducting randomised controlled trials (RCTs) in primary care is challenging; recruiting patients during time-limited or remote consultations can increase selection bias and physical access to patients' notes is costly and time-consuming. We investigated barriers and facilitators to running a more efficient design.

Design: An RCT aiming to reduce antibiotic prescribing among children presenting with acute cough and a respiratory tract infection with a clinician-focused intervention, embedded at the practice-level. By using aggregate-level, routinely collected data for the co-primary outcomes, we removed the need to recruit individual participants.

Setting: Primary care

Participants: Baseline data from GP practices and interviews with individuals from Clinical Research Networks (CRNs) in England who helped recruit practices and Clinical Commission Groups (CCGs) who collected outcome data.

Intervention: The intervention included: i) explicit elicitation of parental concerns, ii) a prognostic algorithm to identify children at low risk of hospitalisation and iii) provision of a printout for carers including safety-netting advice.

Co-Primary outcomes: For 0-9 year olds - i) Dispensing data for amoxicillin and macrolide antibiotics and ii) hospital admission rate for RTI.

Results: We recruited 294 of the intended 310 practices (95%) representing 336,496 registered 0-9 year-olds (5% of all 0-9 year-old children). Included practices were slightly larger, had slightly lower baseline prescribing rates and were located in more deprived areas reflecting the national distribution. Engagement with CCGs and their understanding of their role in this research was variable. Engagement with CRNs and installation of the intervention was straight-forward although the impact of updates to practice IT systems and lack of familiarity required extended support in some practices. Data on the co-primary outcomes was almost 100%.

Conclusions: The infrastructure for trials at the practice level using routinely collected data for primary outcomes is viable in England and should be promoted for primary care research where appropriate.

Trial registration: ISRCTN11405239

For peer review only

Article Summary

<u>Strengths and limitations of this study</u>

- Utilising routinely collected data in primary care at the practice level removes the burden of individual patient recruitment and potential for selection bias.
- Using the NIHR Clinical Research Network across England and Clinical Commissioning Groups (CCGs) allowed recruitment of practices with a broader research experience.
- Embedding the intervention within practice systems utilises existing patient data.
- Engagement with third parties (such as the CCGs) to collect primary outcome data adds another layer of administrative burden.
- Data collected at the practice level are limited (e.g. absence of denominator data such as patients consulting for different conditions) so are only viable if suitable proxy markers can be found for the outcomes of interest.

For peer review only

Introduction

Conducting randomised controlled trials (RCTs) in primary care is essential for the development of a robust evidence base to improve the treatment, health and well-being of patients. However, it is a difficult environment in which to conduct research. In England, general practices are already divided into those who are willing to conduct research and those who are not which challenges the external validity of any successful trial when rolling out an intervention.[1] Effective recruitment strategies generally require practitioner involvement[2] but this is difficult in the time allowed for consultations and can lead to the exclusion of patients for whom recruitment might be more challenging and therefore increase the risk of selection bias.[3] The time pressure can be compounded if the intervention is not fully integrated into the practice computer systems; a stand-alone tool takes time to open and close, may not draw upon information already collected within the system and may be less amenable to modification on a wider scale. In Primary Care research there are also difficulties in collecting patient outcomes. This is largely dependent on physical access to patient notes and is both costly and time-consuming.

Conducting trials at the practice level removes the need for clinicians to recruit individual patients and opens up the possibility of utilising routinely collected data for patient groups at each practice. Nationally collected routine data by practice and patient age are available from Clinical Commissioning Groups (CCGs)[4] and reliable data related to activity or financial transactions, such as the dispensing of medications and hospitalisation rates can, be used for primary trial outcomes. The research infrastructure in England created by the National Institute for Health Research (NIHR) could make the wider recruitment of practices viable.[5] Clinical Research Networks (CRNs) support health and care organisations to be research active[6] and can help recruit practices at a national level, including those serving diverse socioeconomic populations, improving the generalisability of findings.[7] Over 90% of the 7526 GP practices in England use either the *Egton Medical Information Systems* (*EMIS*) (56%) or *SystmOne* (34%) and both have the facility to integrate intervention algorithms and utilise patient data already on the system.[8] Simpler designed studies, which place fewer demands on clinicians and practices compared with other studies, may also encourage research-naïve practices to take part in research.

Recruitment at the patient-level was found to be a challenge in our feasibility study with a significant differential in the health of the patients between arms, increased consultation times due to individual recruitment and using a stand-alone intervention and costly in terms of collecting individual patient notes. [9,10] The on-going main trial, which aims to reduce antibiotic prescribing among children presenting with an upper respiratory tract infection and cough (The CHIldren's COugh or CHICO trial) was re-designed at the practice level.[11] We report on our experience of a

For peer review only - http://bmjopen.bmj.com/site/about/guidelines.xhtml

simpler design; recruiting practices nationally utilising routinely collected data as the primary outcome, integrating the intervention within electronic health record systems and a light-touch approach to data collection. We also used a brief survey to find out how CRNs communicate with practices and semi-structured interviews of key individuals in CRNs and CCGs to look at the barriers and facilitators to this approach.

Methodology

Respiratory tract infections (RTIs) in children are common and present major resource implications for primary care.[12,13] Unnecessary use of antibiotics is associated with the development and proliferation of antimicrobial resistance. In 2016 our 5-year NIHR-funded 'TARGET' programme developed a prognostic algorithm to identify children with acute cough and RTI at very low risk of hospitalisation within 30 days and unlikely to need antibiotics.[14] The intervention included: i) explicit elicitation of parental concerns, ii) the results of the prognostic algorithm accompanied by prescribing guidance and iii) provision of support for a no-antibiotic strategy through a printout for carers including safety netting advice.[15]

The subsequent feasibility study, recruiting at the patient-level, showed prescribing reductions in both arms of the trial but also exposed the differential recruitment of significantly healthier children in the control arm. In the qualitative interviews, clinicians reported preferential recruitment of less unwell children as these were quicker to manage and therefore easier to recruit.[10]

To negate differential recruitment, and conserve resources, an 'efficient design' was proposed for the full trial. The main changes in design were:

i) Recruiting practices (with the help of CRNs and CCGs) rather than individual patients.
ii) Using routinely collected data from CCGs and from national reporting systems for the primary outcome rather than directly from the practices.
iii) Integrating the intervention within electronic medical records (with a triggered pop-up) rather than a stand-alone web-based tool.
iv) A light-touch approach to collecting secondary data using practice champions (eliciting the help of a practice manager or someone familiar with practice systems) rather than accessing patient notes.

The CHICO RCT is an efficient, pragmatic open label, two-arm (intervention vs. usual care) trial with an embedded qualitative study, with randomisation at the practice level, using routine antibiotic dispensing and hospitalisation data to assess effectiveness. The study population is children aged 0-9 years presenting with acute cough and RTI. Oral suspensions are more often given to this age group.

For peer review only

The setting is consultations in primary care practices with prescribing clinicians in diverse regions across England. Recruitment is at the practice level, so consent is not required for individual participants. Recruitment of practices is via CCGs and by using the Clinical Research Network (CRN) who support patients, the public and health and care organisations across England to participate in high-quality research. Research ethics approval was given by London-Camden and Kings Cross Research Ethics Committee (ref:18/LO/0345). This research is funded by the National Institute for Health Research (NIHR) Health Technology Assessment (HTA) programme (funder ref: 16/31/98).

Feedback on the roles of CRNs and CCGs in recruiting practices were obtained from a short questionnaire sent to all CRNs and semi-structured interviews with a convenience sample of key individuals in CRNs and CCGs. The questionnaire focussed on how CRNs communicate with practices and the subsequent interviews explored these points and individual opinions of conducting efficient design trials in primary care in greater depth. The interviews were conducted in September 2021. Questionnaire responses were summarized in a table and pertinent comments highlighted. Interviews were audio-recorded, transcribed verbatim and analysed using a framework approach.[16] Transcripts were all read for familiarisation with the data and an initial sample of transcripts using both deductive (key issues in the interview topic guide), and inductive (derived from the data) codes. The initial codes were discussed with the wider team and a thematic framework was developed and used to code the whole of the dataset. Findings were then summarised in table format and used to inform wider understanding of the facilitators and barriers to conducting CHICO.

Patient and Public Involvement

This intervention has been developed collaboratively with our parent advisory group (PAG) and clinical advisory group (CAG) throughout the 'TARGET' programme. Their comments and suggestions about the format of the intervention and parent/carer materials have informed the intervention and the design of the earlier feasibility study and the design of the main trial.

Results

i) Recruiting practices

The sample size calculation indicated we needed 310 practices (155 intervention, 155 usual care). Between October 2018 and October 2020, we recruited 294 practices (94.8%) representing 336,496 registered patients aged 0-9 years old (5.0% of all 0-9 year-old children in England).[17] Practices were recruited using all 15 CRNs in England and 47 of the 211 CCGs covering the English regions in 2019 (Figure 1).[18] Table 1 gives an indication of the generalisability of the results. Over half of the practices (59%) were larger than the average list size in England, around a third (32%) had higher prescribing

For peer review only

rates than the national average and over a quarter of the practices (26%) were in the most deprived socio-economic quintile reflecting the national distribution of more practices being located in urban areas.

Table 1. Practice baseline characteristics

	n	**CHICO practices (n=294)** n (%) or *Median (IQR)*
Total number of 0-9 year olds listed[a]		339,496
Practice characteristics, using routine data sources		
Above the average list size in England[b]	294	173 (59%)
Median list size of 0-9 year olds (IQR)[a]	294	*984 (678, 1405)*
Above the median prescribing rate in England[c]	294	93 (32%)
Median prescribing rate (IQR)[d]	294	*17.9 (14.3, 24.6)*
Patient characteristics, using a practice questionnaire [e]		
Median # of General Practitioners	290	*6.0 (4.0, 9.0)*
Median # of salaried nurses	238	*2.0 (1.0, 4.0)*
Median # of sessional nurses	133	*0.0 (0.0, 1.0)*
Median # pharmacist independent prescribers	190	*1.0 (0.0, 1.0)*
Median # of Locums in previous 12 months	203	*3.0 (2.0, 6.0)*
Median distance to the nearest childrens A&E (miles)	287	*4.5 (2.2, 10.0)*
IMD[f] quintile based on practice postcode		
1 (most deprived)		77 (26%)
2		60 (20%)
3	294	60 (20%)
4		54 (18%)
5 (least deprived)		43 (15%)
Income Deprivation Affecting Children Index Score[g]		
Median proportion (IQR)	294	*15% (8%, 26%)*

[a] Based on the mean practice list size of 0-9 year olds over the 12-months prior to randomisation, [b] The median practice list size of 0-9 year olds in England in October 2019, the mid-point of recruitment, was 852 patients[14] , [c] Based on amoxicillin and macrolide use in England CCGs (median=22) for all ages[19]. [d] Based on the total number of amoxicillin/macrolide items dispensed over the 12-months prior to randomisation, divided by the practice list size, [e] As reported by the practice champion, [f] index of multiple deprivation, [g] The Income Deprivation Affecting Children Index (IDACI) measures the proportion of all children aged 0-15 living in income deprived families. It is a subset of the Income Deprivation Domain which measures the proportion of the population in an area experiencing deprivation relating to low income. The definition of low income used includes both those that are out-of-work, and those that are in work but who have low earnings (and satisfy the respective means tests).

Assuming most families live close to their GP practice, using Income Deprivation Affecting Children Index (IDACI), we estimate 15% of children included in the trial lived in a deprived neighbourhood (defined as those people that are out-of-work, and those that are in work but who have low earnings). Typically, the number of registered 0-9 year olds was just under 1000 per practice, staffed with a median of 6 general practitioners, 2 salaried nurses, 1 pharmacist and 3 locums over one year. The practice list size of 0-9 year old children ranged from 149 to 6969 with 64 practices (22%) having more than 1500 children registered (Figure 2a). The number of amoxicillin or macrolides dispensed, over the 12-month baseline period prior to randomisation ranged from <5 to >55 per 100 patients (Figure 2b) with a median of 18 items (Table 1). Recruitment was planned over a 12-month period but took 24 months to complete. This in part was due to first having to obtain agreement from CCGs (providing the co-primary outcomes) to participate in the study and delays due to the COVID-19 pandemic.

For peer review only

Facilitators

The 15 CRNs were helpful with the recruitment of practices (Table 2); presenting our study at CRN meetings and using the national CRN lead for support helped facilitate a co-ordinated national approach.

Table 2. Semi-structured interviews with members of CRNS and CCG

Question	Response
What are the facilitators to practice recruitment?	*"Communicating with the practices. I think in an academic centre, if you're writing to practices, they don't really know you, do they? Whereas actually, if we put it in our local bulletins and keep encouraging practices to think about it, then it's probably a better way of increasing uptake."* [P4 CCG]<br><br>*"There's been a nice friendship [with study team] and trying to create the right words to share with practices."* [P1 CCG]<br><br>*"Just keep on plugging. I think quite often they're either going through a really busy time, I've contacted the wrong person or there's not enough information there for them to make a snap decision. The more information I've got and the simpler I make it, then the more likely they are to say yes or no."* [P3 CRN]
What are the barriers to practice recruitment?	*"To be honest it was a little bit more difficult to get the paperwork signed by the CCG as well within the practices, 'cause a lot of the practices we generally have a named contact, someone who already knows us and we've got relationships with…so it was a lot easier to get engagement than it was from CCGs."* [P2 CRN]<br><br>*"I try to avoid the practice managers making the decisions if I can because they are good gatekeepers. Well our practice is very busy, that GP won't be interested when actually sometimes they quite often are."* [P3 CRN]
Tell us about Research active and non-active (naïve) practices?	*"It's usually the research active sites that get back to us but sometimes others do respond and want to take part in studies and we've got this ongoing engagement programme with all the practices in ((city)) to try and get more of them on board with research even if it's just doing simple stuff."* [P5 CRN]<br><br>*"I would say that about a third of the practices were what I would call research naïve or inexperienced, green as in they hadn't really had a sort of established relationship with us in the past."* [P2 CRN]
Tell us about the role of CCGs in Research?	*"I mean in reality they [CCG] don't play a major part in – and never actually have a major role in research. It's not a core business of a CCG like it would be in a provider. So we don't have a research department, which is probably why the CHICO trial ended up at my door because it was about prescribing."* [P4 CCG]
Tell us about efficient design trials?	*"Yeah, go for it because once you've got the practice on board, it's almost like they don't have to do as much either, I would definitely encourage practices to take on this type of research, definitely, as long as you've got everybody on board and it all works through then yeah, there's no reason why not."* [P3 CRN]
	"It seems quite suited to primary care because I think primary care's biggest issue is time. So, if they've got a patient in front of them, the chance of them actually getting that patient to consent is probably quite low because it's just they – that's an extra minute on a ten-minute appointment isn't it really?" [P4 CCG]

Feedback from 11 of the 15 CRNs indicated that some contact practices that opt-in to conduct research whilst others contact all the practices in their area; around half the practices had joined

For peer review only

the Research Sites Initiative scheme (NIHR funded scheme to enable research delivery) and were often the first to be contacted. Recruitment via CCGs had a wider reach of practices than via CRNs, although CCG participation in recruitment was more variable depending on capacity. Using quarterly study newsletters for practices, CRNs and CCGs with league tables monitoring levels of recruitment improved responses.

Barriers

Limiting the contact to practices that want to opt-in to research via some of the CRNs misses the opportunity of letting research-naïve practices know about light-touch efficient design studies. The level of engagement from CRNs varied slightly but much more between CCGs. Some CCGs were averse to getting involved in research or cited lack of capacity as a reason to be excluded from the research (Table 2). It was difficult to know which individuals to contact and how to do so (some CCGs did not appear to be public-facing), response times were often slow, their role in research was often misunderstood, staff changes hindered communication and a number of CCGs merged during the study period. At the start of recruitment, October 2018, there were 211 CCGs across England. By the end of follow up, September 2021, there were only 106 CCGs. While this did reduce the number of CCG contacts required to obtain the data, it sometimes resulted in change of staff who were not familiar with the trial or its requirements. Of the 294 practices we recruited, we are aware of at least 22 practices (7%) who merged during their baseline/follow up annual data capture. We excluded practices who anticipated a merger with another practice but had no control over this once the practice was randomised, especially in the rare instances when the merging practices were randomised to different arms of the trial. The length of time from expression of interest from practices to randomisation was longer than expected due to the delayed site agreements returned from the practices.

<u>ii) Using routinely collected data for the primary outcomes</u>

The co-primary outcomes in the trial were i) practice dispensed prescription data for amoxicillin and macrolide antibiotics for children 0-9 years and ii) hospital admission rate for RTI among 0-9 year olds. The antibiotic dispensing rates were retrieved from the *NHSBSA ePACT2* reporting system for all practices in the trial and practice list size data by 5 year epoch was retrieved from *NHS Digital* and combined to create a dispensed prescription rate for amoxicillin and for macrolide antibiotics.[20] The hospital admission rates are routinely collected by all English CCGs. The ascertainment of these data was 99.7% (293/294) for antibiotic dispensing and hospitalisation rates. Data for one practice was lost due to a merger.

For peer review only

Facilitators

Using aggregated data avoids the need for the consulting physician to solicit individual consent, reduces or eliminates the risk of selection bias and removes the task of accessing individual patient notes. Routine data are often collected monthly so data completeness and quality can be monitored throughout the collection period, which was particularly important in this trial to scrutinise and report any sudden changes in hospital admission rates to the Data Monitoring Committee. The routinely collected data can be imported to data management software, thus there is less likelihood of data entry errors and missing data are rare. The cost of research staff and time taken to collect the primary outcome data was much reduced compared to a traditional trial. In total 11/294 practices asked to be withdrawn from the study (3.7%) and 14/294 (4.8%) were lost to follow-up from the study; the main reasons cited being lack of capacity and prioritising the COVID-19 pandemic. A further advantage of collecting routine data is that withdrawal of a practice does not necessarily mean a loss of the primary outcome data for that practice (if the data are already in the public domain).

Barriers

A potential problem with aggregate data collected from a third party is where data are suppressed, owing to a low number of events, although in this study data were collected over a 12-month period largely avoiding this problem. Some liaison was needed between the trial team and the CCGs to know exactly what data were available and in what format this could be presented. Dispensed prescription data, practice list size data and hospital admission data are reliable as they feed into financial transactions, however data reporting emergency department [ED] attendance were less so due to a limited coding set. Many ED attendances do not have a diagnosis coded, therefore the number of attendances attributable to RTI is likely to be inaccurate.

For peer review only

iii) Integrating the intervention within the electronic medical record system

The trial only included practices using the *EMIS* system. The practice champion was given written instructions on how to install the intervention within *EMIS*. Self-directed training materials were provided for all clinicians using the intervention. The algorithm consists of seven predictors of future hospitalisation, two of which were already in the practice systems (child age and history of asthma) and thus pulled into the algorithm automatically from patient records, and five which were entered during consultation (illness duration, raised temperature, vomiting in last 24 hours, presence of wheeze and presence of intercostal or subcostal recession).[21] Carer concerns were elicited during the consultation and formed part of a personalised leaflet generated from the system containing an easy to read 'Caring for Children with Coughs' graphic and safety-netting advice about when to seek

medical care or advice.[16] Intervention use was monitored using searches on the *EMIS* system, run by the practice champion and shared with the research team.

Facilitators

Installation of the intervention was relatively straight-forward for *EMIS*. Practice systems allow for user-friendly manipulation so interventions can be integrated and interface with system data already collected, thus negating any additional clinical workload. Screen pop-ups can also be used to notify clinicians of eligible patients to the study.

Barriers

Familiarity with the *EMIS* system varied between practices thus the level of support required from the research team to help install the intervention and download usage also varied. Provision to help install or use third-party algorithms is not offered by system providers. EMIS upgrades to the system during the trial meant that rewriting installation instructions, resources and testing had to be carried out and fed back to the practices. For instance, the READ codes used to identify clinical terms within consultations were upgraded by EMIS in the early months of 2020 which meant the algorithm had to be amended so the intervention would function correctly. We found that our funded provision of IT support throughout the study was crucial to the smooth running of the integrated intervention. Although pop-ups can be used, some clinicians found them irritating and switched them off whilst other practices had so many pop-ups (especially during the COVID-19 pandemic) that the CHICO pop-up was often obscured.

<u>iv) A light-touch approach to data collection</u>

Clinicians were not required to provide any data about individual participants (apart from reporting serious adverse events), those in the intervention group were asked to familiarise themselves with the tool and use it, whilst those in the control arm were asked to provide usual care. Data collection from the practices directly was limited to short baseline and follow-up questionnaires. Data on intervention usage were downloaded from *EMIS* and the co-primary outcome downloads from ePACT2 and the relevant CCGs.

Facilitators

The light touch approach reduced the time needed during consultation to record information, the trawling of patient notes and provided a more objective data resource downloaded from the system rather than from individual input. Practice champions, familiar with practice systems, played an important role in obtaining the required data. Baseline questionnaires were received from 294/294 practices (100%), follow-up questionnaires were received from 265/294 practices (90%) whilst

For peer review only

intervention usage was collected from 116/144 of the intervention practices (81%), indicating the data burden was not too onerous.

Barriers

Conversely, this approach reduced the level of interpretation that can be gleaned from the data. Removing patient level recruitment results in a loss of denominator data, in our case not knowing how many individual children consulted for RTI and cough. As a proxy we used the number of children registered at each practice. Given the diversity of the practices included in the trial we are assuming that those children taking part in the trial were no different from children in the general population, but we have no way of testing this assumption. We also lost detail that may be taken from the consultation of whether antibiotics were prescribed immediately or delayed relying instead on the number of antibiotics being dispensed. The lack of contact with usual care practices (apart from reminders to provide SAE reports) runs the risk of practices wanting to withdraw from the study, especially with changes of staff. Consideration also needs to be given to the ethical implications of not seeking consent from individual patients.

Discussion

The National Institute for Health Research (NIHR) is keen to see the design, development and delivery of more efficient, faster, innovative studies which provide robust evidence to inform clinical practice and policy.[22] The CHICO study demonstrates that an efficiently designed practice-level large trial in primary care using routinely collected data is feasible and potentially good value for money. The average cost of an HTA RCT was £1.25 million in 2019/20,[23] whereas the cost of the CHICO RCT, which included over 300,000 children (5% of the entire national 0-9 year-old population) and 4% of all GP practices in England, was below £1 million. Utilising routinely collected data as the primary outcome reduces problems with missing data whilst removing the burden of patient recruitment and focusing the clinician's time on using the intervention reflects real life practice. These findings are pertinent to the health care system in England but might lend themselves to similar primary care networks in other countries.

Integrating the intervention within the practice system both exploits the data already available and adds to the patient's record avoiding duplicating of effort and saves time. Primary care practices are often very busy and the average length of face-to-face consultation in the UK is around 10 minutes; less than half the time given to patients in, for example, Sweden and the US.[24] Growing demands on

For peer review only

primary care services have also led to policymakers promoting telephone and video consultations, even before the COVID-19 pandemic, and these sometimes don't lend themselves to enlisting patients in research.[25] Reducing the research burden for participants and clinicians is always desirable, but particularly so given the increasing time constraints in primary care. This light-touch approach may also be more appealing for practices who maybe research naïve; investing in different recruitment strategies[26] using existing networks could potentially yield a more representative sample than previous trials in primary care from which to generalise any findings. The design of the CHICO trial retrospectively scored highly in each domain of the PRECIS-2 ((PRagmatic Explanatory Continuum Indicator Summary) tool[27] suggesting it is a pragmatic randomised trial focusing on delivery in the "real world" rather than providing the best chance to demonstrate a beneficial effect in an idealised setting.

Efficient design studies which utilise routine data and recruit at practice level face their own challenges. First, this approach would not be suitable if individual patient consent is required or for trials using a patient reported outcome as the primary outcome, as these are not widely collected in routine care. Furthermore, the number of CCGs in England almost halved during the study period due to organisational restructuring which made it difficult to administer the trial. From March 2022 Integrated Care Systems will replace CCGs and this will provide new bureaucracy for researchers to develop relationships with. There is a lack of uniformity when approaching these commissioning groups and their role in research needs clarifying.[28] Using a convenience sample the qualitative interviews have limited insight but suggest research needs to be higher on the agenda. CRNs are more research-focused and can help with recruitment although adoption of the study needs to be made more explicit and a consistent national approach to include research naïve practices needs to be adopted within this network.

If practices are being used as the unit of analysis, then the commitment to research they have signed up for needs to be strengthened. Around 2.5% practices close or merge each year in England whilst some new practices open and recognition of the current research portfolio needs to be part of this process.[29] We were also surprised by the wide variability in practice list size, 8% of practices in the study having 3 sites or more. This has implications for future trials in terms of factoring in variable list sizes for sample size calculations and checking that multiple sites use the same electronic record systems. A light touch approach is only viable if suitable primary outcomes can be identified but makes fidelity more difficult to measure. Losing denominator data such as patients consulting for different conditions will depend on the hypothesis being tested and needs to strike a balance

For peer review only

between accuracy of what you are trying to measure and whether a proxy marker will deliver the population impact of the intervention. Using the patient list size of 0-9 year olds as a proxy denominator instead of those consulting for RTI accounts for the variability between the size of different practices but not necessarily the disease burden.

If one of the main intentions of primary care research is to provide the clinician with better tools, then we need to work more closely with those who supply the toolbox (IT system providers) and the third parties who provide the data within which practices work. The infrastructure to conduct efficiently designed trials that do not require patient-reported outcomes in England is potentially viable but does require more investment in time and effort to make recruitment of practices and data collection more accessible to researchers.

Authors Contributions

AH, PB, PL, NF and JI were responsible for developing the research questions. PB, AH, JI, PL, CCab, CClem, EB, MG, JH, SC, AL and NF and are responsible for the study design and collection of data. PS, JT and SB are responsible for study management and coordination. GY, PD and CClem are responsible for the analysis of the data. PB drafted the paper. All authors read, commented on and approved the final manuscript.

Acknowledgements

This study was designed and delivered in collaboration with the Bristol Randomised Trials Collaboration (BRTC), part of the Bristol Trials Centre, is in receipt of National Institute for Health Research CTU support funding. The University of Bristol is acting as the sponsor for this trial and the trial is hosted by the NHS Bristol, North Somerset and South Gloucestershire Clinical Commissioning Group (CCG). The authors would like to thank all General practices, CCGs and CRNs for their involvement in CHICO. The authors would also like to thank the members of the TSC and DMC.

Funding

This research is funded by the National Institute for Health Research (NIHR) Health Technology Assessment (HTA) programme (funder ref: 16/31/98). The views expressed are those of the authors and not necessarily those of the NIHR or the Department of Health and Social Care.

For peer review only

Competing interest

None declared

Data Sharing

The data are obtained from a third party (Clinical Commissioning Groups and Public Health England) and are not publicly available

Ethics Approval

Research ethics approval was given by London-Camden and Kings Cross Research Ethics Committee (ref:18/LO/0345).

For peer review only

References

1) Allcott H. Site Selection Bias in Program Evaluation. *The Quarterly Journal of Economics* August 2015;130 (3): 1117–1165.
2) Ngune I, Jiwa M, Dadich A, Lotriet J, Sriram D. Effective recruitment strategies in primary care research: a systematic review. *Qual Prim Care* 2012;20(2):115-23
3) Foster JM, Sawyer SM, Smith L, Reddel HK, Usherwood T. Barriers and facilitators to patient recruitment to a cluster randomized controlled trial in primary care: lessons for future trials. *BMC Med Res Methodol* 2015 Mar 12;15:18
4) Clinical Commissioning Groups https://www.england.nhs.uk/ccgs/
5) National Institute for Health Research https://www.nihr.ac.uk/researchers/collaborations-services-and-support-for-your-research/
6) Clinical Research Network https://www.nihr.ac.uk/explore-nihr/support/clinical-research-network.htm
7) Bower P, Grigoroglou C, Anselmi L, Kontopantelis E, Sutton M, Ashworth M, Evans P, Lock S, Smye S, Abel K. Is health research undertaken where the burden of disease is greatest? Observational study of geographical inequalities in recruitment to research in England 2013-2018. *BMC Med* 2020 May 18;18(1):133.
8) Kontopantelis E, Stevens RJ, Helms PJ, Edwards D, Doran T, Ashcroft DM. Spatial Distribution of Clinical Computer Systems in Primary Care in England in 2016 and Implications for Primary Care Electronic Medical Record Databases: A Cross-Sectional Population Study. *BMJ Open* 2018 Feb 28;8(2):e020738
9) Turnbull SL, Redmond NM, Lucas P, Cabral C, Ingram J, Hollinghurst S, Hay AD, Peters TJ, Horwood J, Little P, Francis N, Blair PS. The CHICO (Children's Cough) Trial protocol: a feasibility randomised controlled trial investigating the clinical and cost-effectiveness of a complex intervention to improve the management of children presenting to primary care with acute respiratory tract infection. *BMJ Open* 2015 Sep 15;5(9):e008615.
10) Blair PS, Turnbull S, Ingram J, Redmond N, Lucas PJ, Cabral C, Hollinghurst S, Dixon P, Peters T, Horwood J, Little P, Francis NA, Gilbertson A, Jameson C, Hay AD. Feasibility cluster randomised controlled trial of a within-consultation intervention to reduce antibiotic prescribing for children presenting to primary care with acute respiratory tract infection and cough. *BMJ Open* 2017 May 9;7(5): e014506.
11) Seume P, Bevan S, Young G, Ingram J, Clement C, Cabral C, Lucas PJ, Beech E, Taylor J, Horwood J, Dixon P, Gulliford M, Francis NA, Creavin S, Lane AJ, Hay A, Blair PS. Protocol for an 'efficient design' cluster randomised controlled trial to evaluate a complex intervention to improve antibiotic prescribing for CHIldren presenting to primary care with acute COugh and respiratory tract infection: The CHICO study. *BMJ Open.* 2021 Mar 29;11(3): e041769.
12) Smith DRM, Dolk FCK, Pouwels KB, Christie M, Robotham JV, Smieszek T. Defining the appropriateness and inappropriateness of antibiotic prescribing in primary care. *J Antimicrob Chemother* 2018 Feb 1;73(suppl_2): ii11-ii18.
13) Vodicka TA, Thompson M, Lucas P, Heneghan C, Blair PS, Buckley DI, Redmond N and Hay AD. Reducing antibiotic prescribing for children with respiratory tract infections in primary care: a systematic review. *Br J Gen Pract* 2013 Jul;63(612): e445-54.
14) Hay AD, Redmond NM, Turnbull S, Christensen H, Thornton H, Little P, Thompson M, Delaney B, Lovering AM, Muir P, Leeming JP, Vipond B, Stuart B, Peters TJ, Blair PS. Development and internal validation of a clinical rule to improve antibiotic use in children

For peer review only

presenting to primary care with acute respiratory tract infection and cough: a prognostic cohort study. *Lancet Respir Med* 2016 Nov;4(11):902-910.

15) Lucas PJ, Ingram J, Redmond NM, Cabral C, Turnbull SL, Hay AD. Development of an intervention to reduce antibiotic use for childhood coughs in UK primary care using critical synthesis of multi-method research. *BMC Med Res Methodol*. 2017 Dec 28;17(1):175.
16) Ritchie J, Spencer L. Qualitative data analysis for applied policy research. In: A. Bryman and R. G. Burgess, ed. Analyzing qualitative data1994:173-94.
17) Population estimate of 0-9 year olds in England using October 2019 (mid-point of recruitment) https://digital.nhs.uk/data-and-information/publications/statistical/patients-registered-at-a-gp-practice/october-2019
18) CCG annual assessment 2019/20 © NHS England and NHS Improvement 2020 Publication approval reference: PAR112 https://www.england.nhs.uk/wp-content/uploads/2020/11/ccg-annual-assessment-report-19-20.pdf
19) Estimate of antibiotic items prescribed per CCG (October 2017 to September 2018) https://openprescribing.net/analyse/#org=CCG&orgIds=15E,15C,D2P2L,14Y,06H,04Y,27D,15N,01A,05D,97R,D4U1Y,11M,D9Y0V,06N,91Q,11N,15F,99A,14L,01K,13T,06T,05G,W2U3Z,52R,10Q,01G,05Q,11X,M2L0M,05W,72Q,36L,05V,00P,92A,03Q&numIds=0501013B0,5.1.5&denom=total_list_size&selectedTab=summary
20) ePACT2 prescribing data held by NHS Prescription Services. https://www.nhsbsa.nhs.uk/epact2
21) Hay AD, Redmond NM, Turnbull S, Christensen H, Thornton H, Little P, Thompson M, Delaney B, Lovering AM, Muir P, Leeming JP, Vipond B, Stuart B, Peters TJ, Blair PS. Development and internal validation of a clinical rule to improve antibiotic use in children presenting to primary care with acute respiratory tract infection and cough: a prognostic cohort study. *Lancet Respir Med*. 2016 Nov;4(11):902-910.
22) NIHR: Annual Efficient Studies funding calls for CTU projects. https://www.nihr.ac.uk/documents/ad-hoc-funding-calls-for-ctu-projects/20141
23) NIHR Annual Report 2019/20. Department of Health & Social Care (page 5) https://www.nihr.ac.uk/documents/about-us/our-contribution-to-research/research-performance/NIHR_Annual_Report_19_20.pdf
24) Irving G, Neves AL, Dambha-Miller H, Oishi A, Tagashira H, Verho A, Holden J. International variations in primary care physician consultation time: a systematic review of 67 countries. *BMJ Open* 2017 Nov 8;7(10):e017902
25) Hammersley V, Donaghy E, Parker R, McNeilly H, Atherton H, Bikker A, Campbell J, McKinstry B. Comparing the content and quality of video, telephone, and face-to-face consultations: a non-randomised, quasi-experimental, exploratory study in UK primary care. *Br J Gen Pract* 2019 Aug 29;69(686):e595-e604
26) Ellis SD, Bertoni AG, Bonds DE, Randall Clinch C, Balasubramanyam A, Blackwell C, Chen H, Lischke M, Goff Jr DC. Value of recruitment strategies used in a primary care practice-based trial. *Contemp Clin Trials* 2007 May;28(3):258-67.
27) Loudon K, Treweek S, Sullivan F, Donnan P, Thorpe KE, Zwarenstein M. The PRECIS-2 tool: designing trials that are fit for purpose. *BMJ*. 2015 May 8;350:h2147
28) Checkland K, McDermott I, Coleman A, Perkins N. Complexity in the new NHS: longitudinal case studies of CCGs in England. *BMJ Open* 2016 Jan 7;6(1):e010199
29) Practices closing or merging

For peer review only

https://digital.nhs.uk/data-and-information/find-data-and-publications/supplementary-information/2018-supplementary-information-files/number-of-gp-practices-closed-or-merged-in-england-between-1-july-2016-and-31-december-2017-by-nhs-geographies

For peer review only

Figure 1. The CCGs taking part in the CHICO study (bold), shaded according to the number of items of amoxicillin and macrolides, per 1000 list size at the mid point of recruitment (Oct ’19).

Figure 2a. The distribution of practice list sizes*, of 0-9 year olds, for those practices taking part in the CHICO study

Figure 2b. The distribution of practice dispensing rates*at baseline, for 0-9 year olds, for those practices taking part in the CHICO study

For peer review only

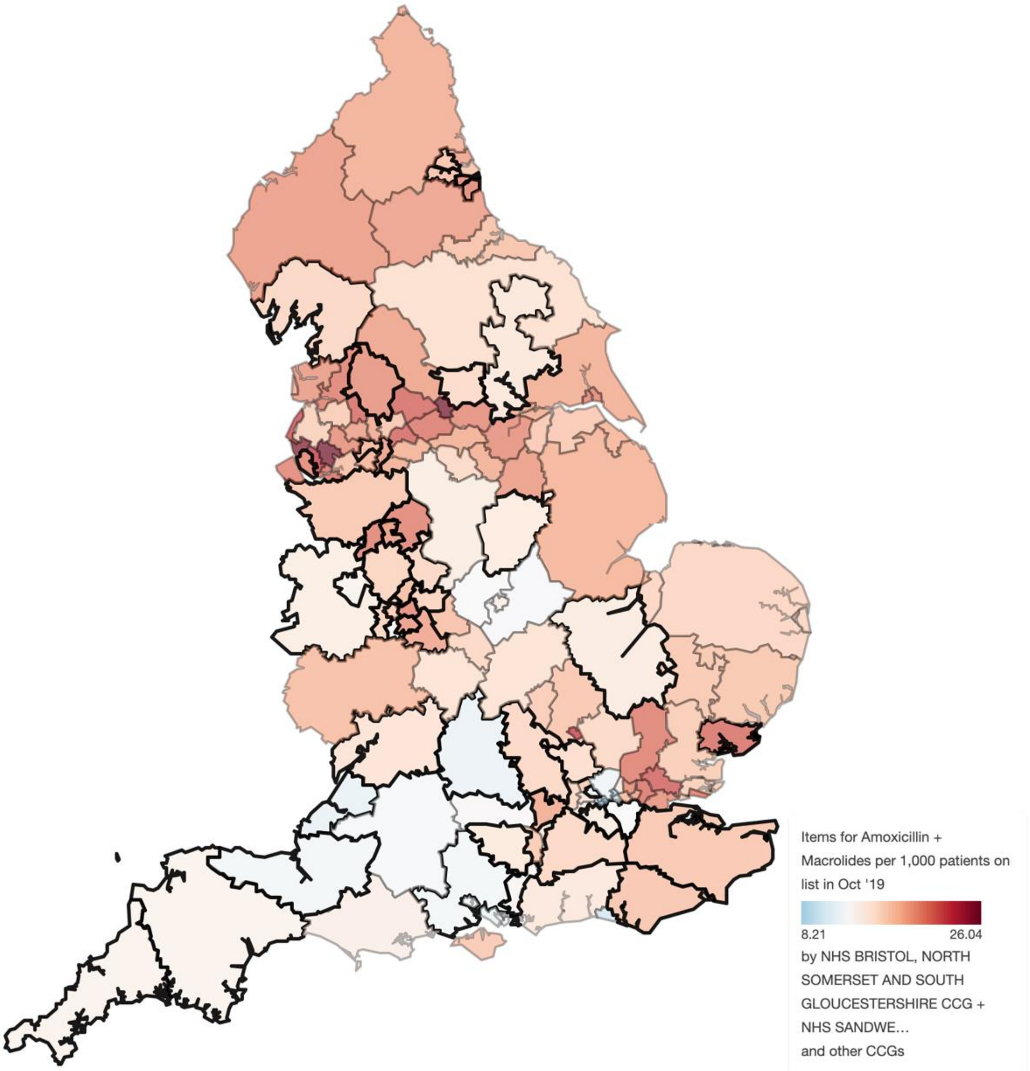

Source: OpenPrescribing.net, EBM DataLab, University of Oxford, 2017

For up to date data please refer to:
[https://openprescribing.net/analyse/#org=CCG&orgIds=15E,15C,D2P2L,14Y,06H,04Y,27D,15N,01A,05D,97R,D4U1Y,11M,D9Y0V,06N,91Q,11N,15F,99A,14L,01K,13T,06T,05G,W2U3Z,52R,10Q,01G,05Q,11X,M2L0M,05W,72Q,36L,05V,00P,92A,03Q&numIds=0501013B0,5.1.5&denom=total_list_size&selectedTab=map]

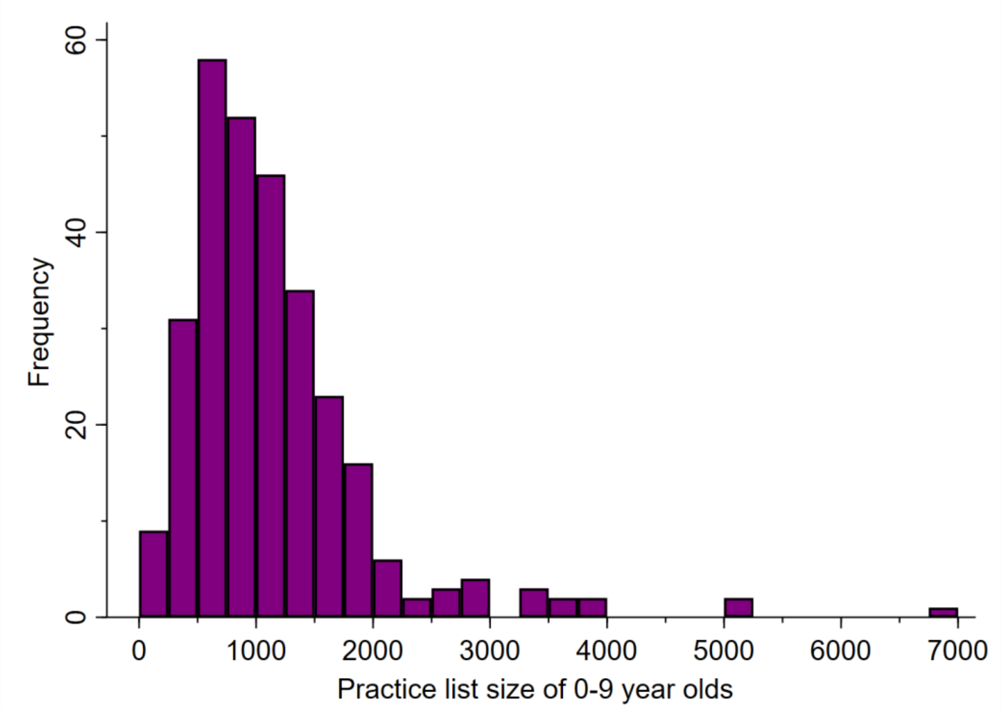

*Defined for each practice as the mean list size of 0-9 year olds, over the 12 months prior to randomisation (source: https://digital.nhs.uk/data-and-information/publications/statistical/patients-registered-at-a-gp-practice)

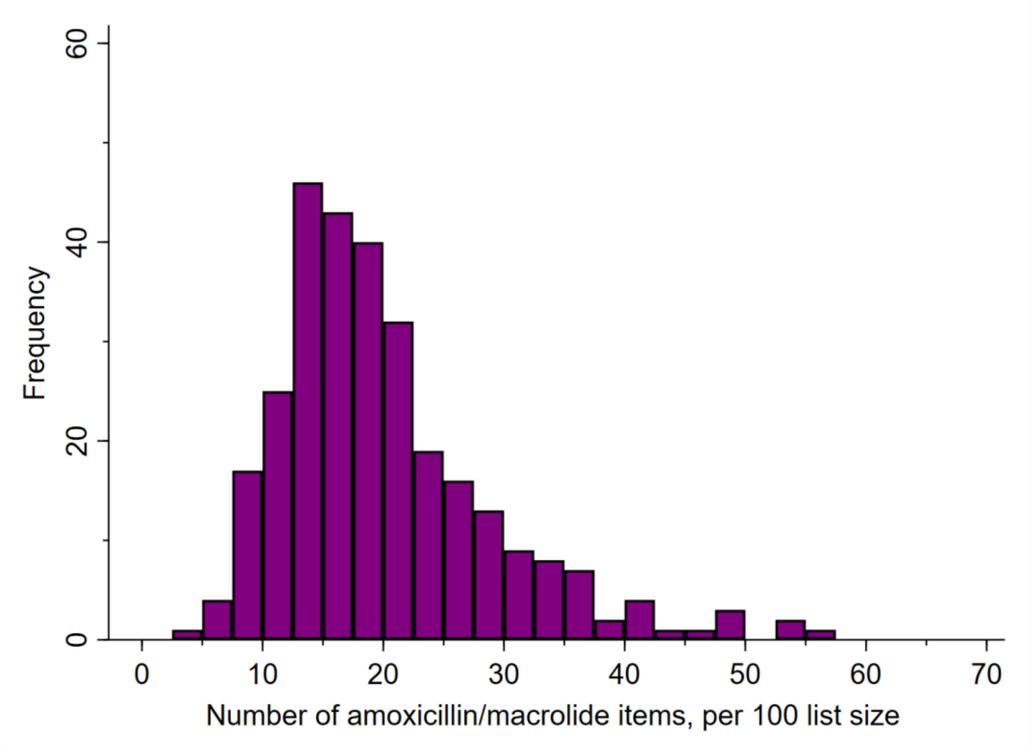

*Defined for each practice as the number of amoxicillin or macrolides dispensed, over the 12-month baseline period prior to randomisation, divided by the practice list size. This may include multiple items per child.

CONSORT 2010 checklist of information to include when reporting a randomised trial*

Section/Topic	Item No	Checklist item	Reported on page No
**Title and abstract**			
	1a	Identification as a randomised trial in the title	1
	1b	Structured summary of trial design, methods, results, and conclusions (for specific guidance see CONSORT for abstracts)	2
**Introduction**			
Background and objectives	2a	Scientific background and explanation of rationale	4-5
	2b	Specific objectives or hypotheses	4-5
**Methods**			
Trial design	3a	Description of trial design (such as parallel, factorial) including allocation ratio	5
	3b	Important changes to methods after trial commencement (such as eligibility criteria), with reasons	N/A^
Participants	4a	Eligibility criteria for participants	N/A^
	4b	Settings and locations where the data were collected	5
Interventions	5	The interventions for each group with sufficient details to allow replication, including how and when they were actually administered	5
Outcomes	6a	Completely defined pre-specified primary and secondary outcome measures, including how and when they were assessed	N/A^
	6b	Any changes to trial outcomes after the trial commenced, with reasons	N/A^
Sample size	7a	How sample size was determined	N/A^
	7b	When applicable, explanation of any interim analyses and stopping guidelines	N/A^
Randomisation:			
Sequence generation	8a	Method used to generate the random allocation sequence	N/A^
	8b	Type of randomisation; details of any restriction (such as blocking and block size)	N/A^
Allocation concealment mechanism	9	Mechanism used to implement the random allocation sequence (such as sequentially numbered containers), describing any steps taken to conceal the sequence until interventions were assigned	N/A^
Implementation	10	Who generated the random allocation sequence, who enrolled participants, and who assigned participants to interventions	N/A^
Blinding	11a	If done, who was blinded after assignment to interventions (for example, participants, care providers, those	N/A^

For peer review only - http://bmjopen.bmj.com/site/about/guidelines.xhtml

		assessing outcomes) and how	
	11b	If relevant, description of the similarity of interventions	N/A^
Statistical methods	12a	Statistical methods used to compare groups for primary and secondary outcomes	N/A^
	12b	Methods for additional analyses, such as subgroup analyses and adjusted analyses	N/A^
**Results**			
Participant flow (a diagram is strongly recommended)	13a	For each group, the numbers of participants who were randomly assigned, received intended treatment, and were analysed for the primary outcome	6
	13b	For each group, losses and exclusions after randomisation, together with reasons	9-10
Recruitment	14a	Dates defining the periods of recruitment and follow-up	6
	14b	Why the trial ended or was stopped	N/A
Baseline data	15	A table showing baseline demographic and clinical characteristics ~~for each group~~	7
Numbers analysed	16	For each group, number of participants (denominator) included in each analysis and whether the analysis was by original assigned groups	N/A^
Outcomes and estimation	17a	For each primary and secondary outcome, results for each group, and the estimated effect size and its precision (such as 95% confidence interval)	N/A^
	17b	For binary outcomes, presentation of both absolute and relative effect sizes is recommended	N/A^
Ancillary analyses	18	Results of any other analyses performed, including subgroup analyses and adjusted analyses, distinguishing pre-specified from exploratory	N/A^
Harms	19	All important harms or unintended effects in each group (for specific guidance see CONSORT for harms)	N/A^
**Discussion**			
Limitations	20	Trial limitations, addressing sources of potential bias, imprecision, and, if relevant, multiplicity of analyses	9-14 (barriers)
Generalisability	21	Generalisability (external validity, applicability) of the trial findings	N/A^
Interpretation	22	Interpretation consistent with results, balancing benefits and harms, and considering other relevant evidence	N/A^
**Other information**			
Registration	23	Registration number and name of trial registry	2
Protocol	24	Where the full trial protocol can be accessed, if available	16
Funding	25	Sources of funding and other support (such as supply of drugs), role of funders	6

^Not applicable for this manuscript, where the pros/cons of the trial design are presented. For more information on these items, please refer to the protocol: 10.1136/bmjopen-2020-041769

*We strongly recommend reading this statement in conjunction with the CONSORT 2010 Explanation and Elaboration for important clarifications on all the items. If relevant, we also recommend reading CONSORT extensions for cluster randomised trials, non-inferiority and equivalence trials, non-pharmacological treatments, herbal interventions, and pragmatic trials. Additional extensions are forthcoming: for those and for up to date references relevant to this checklist, see www.consort-statement.org.

For peer review only - http://bmjopen.bmj.com/site/about/guidelines.xhtml

BMJ Open

Can primary care research be conducted more efficiently using routinely reported practice-level data: a cluster randomised controlled trial conducted in England?

Journal:	*BMJ Open*
Manuscript ID	bmjopen-2022-061574.R2
Article Type:	Original research
Date Submitted by the Author:	15-May-2022
Complete List of Authors:	Blair, Peter; University of Bristol, Population Health Sciences<br>Ingram, Jenny; University of Bristol, Bristol Medical School<br>Clement, Clare; University of Bristol, Bristol Medical School<br>Young, Grace; University of Bristol, Bristol Medical School; Bristol Randomised Trials Collaboration, University of Bristol<br>Seume, Penny; University of Bristol, Centre for Academic Primary Care, Bristol Medical School: Population Health Sciences<br>Taylor, Jodi; University of Bristol, Bristol Medical School<br>Cabral, Christie; University of Bristol, Centre for Academic Primary Care<br>Lucas, Patricia; University of Bristol, School for Policy Studies<br>Beech, Elizabeth; NHS Improvement<br>Horwood, Jeremy; University of Bristol, NIHR ARC West<br>Dixon, Padraig; Oxford University<br>Gulliford, Martin; King's College London, UK<br>Francis, Nick; University of Southampton, School of Primary Care Population Sciences and Medical Education<br>Creavin, Sam T; University of Bristol, Bristol Medical School<br>Lane, Athene; University of Bristol, School of Social and Community Medicine<br>Bevan, Scott; University of Bristol, Centre for Academic Primary Care, Bristol Medical School: Population Health Sciences<br>Hay, Alastair; University of Bristol, Centre for Academic Primary Care
**Primary Subject Heading**:	General practice / Family practice
Secondary Subject Heading:	Respiratory medicine
Keywords:	PRIMARY CARE, Respiratory infections < THORACIC MEDICINE, QUALITATIVE RESEARCH

SCHOLARONE™
Manuscripts

I, the Submitting Author has the right to grant and does grant on behalf of all authors of the Work (as defined in the below author licence), an exclusive licence and/or a non-exclusive licence for contributions from authors who are: i) UK Crown employees; ii) where BMJ has agreed a CC-BY licence shall apply, and/or iii) in accordance with the terms applicable for US Federal Government officers or employees acting as part of their official duties; on a worldwide, perpetual, irrevocable, royalty-free basis to BMJ Publishing Group Ltd ("BMJ") its licensees and where the relevant Journal is co-owned by BMJ to the co-owners of the Journal, to publish the Work in this journal and any other BMJ products and to exploit all rights, as set out in our licence.

The Submitting Author accepts and understands that any supply made under these terms is made by BMJ to the Submitting Author unless you are acting as an employee on behalf of your employer or a postgraduate student of an affiliated institution which is paying any applicable article publishing charge ("APC") for Open Access articles. Where the Submitting Author wishes to make the Work available on an Open Access basis (and intends to pay the relevant APC), the terms of reuse of such Open Access shall be governed by a Creative Commons licence – details of these licences and which Creative Commons licence will apply to this Work are set out in our licence referred to above.

Other than as permitted in any relevant BMJ Author's Self Archiving Policies, I confirm this Work has not been accepted for publication elsewhere, is not being considered for publication elsewhere and does not duplicate material already published. I confirm all authors consent to publication of this Work and authorise the granting of this licence.

For peer review only

Can primary care research be conducted more efficiently using routinely reported practice-level data: a cluster randomised controlled trial conducted in England?

Peter S Blair[1], Jenny Ingram[1], Clare Clement[2], Grace Young[2], Penny Seume[3], Jodi Taylor[2], Christie Cabral[3], Patricia J Lucas[4], Elizabeth Beech[5], Jeremy Horwood[3], Padraig Dixon[8], Martin Gulliford[6], Nick A Francis[7], Sam Creavin[3], J Athene Lane[2], Scott Bevan[2] & Alastair D Hay[3]

Author Affiliations

[1] Centre for Academic Child Health, Bristol Medical School, Population Health Sciences, University of Bristol

[2] Bristol Trials Centre (Bristol Randomised Trial Collaboration), Bristol Medical School, University of Bristol

[3] Centre for Academic Primary Care, Bristol Medical School: Population Health Sciences, University of Bristol.

[4] School for Policy Studies, University of Bristol

[5] NHS England and NHS Improvement South West

[6] Kings College, University of London

[7] Primary Care Research Centre, School of Primary Care Population Sciences and Medical Education, University of Southampton

[8] Nuffield Department of Primary Care Health Sciences, University of Oxford

Corresponding author:

Prof Peter S Blair (Professor of Epidemiology and Statistics), Centre for Academic Child Health, University of Bristol.

Address: Level D, St Michael's Hospital, Southwell St, Bristol, BS2 8EG

Phone: 01173425145 Fax: 01173425154   E-mail: p.s.blair@bris.ac.uk

Word Count (Main text): 3854 words (excluding tables)

Key Words: Respiratory Tract Infection, Antibiotic Prescribing, Primary Care, Efficient Design

For peer review only

Abstract

Objectives: Conducting randomised controlled trials (RCTs) in primary care is challenging; recruiting patients during time-limited or remote consultations can increase selection bias and physical access to patients' notes is costly and time-consuming. We investigated barriers and facilitators to running a more efficient design.

Design: An RCT aiming to reduce antibiotic prescribing among children presenting with acute cough and a respiratory tract infection with a clinician-focused intervention, embedded at the practice-level. By using aggregate-level, routinely collected data for the co-primary outcomes, we removed the need to recruit individual participants.

Setting: Primary care

Participants: Baseline data from GP practices and interviews with individuals from Clinical Research Networks (CRNs) in England who helped recruit practices and Clinical Commission Groups (CCGs) who collected outcome data.

Intervention: The intervention included: i) explicit elicitation of parental concerns, ii) a prognostic algorithm to identify children at low risk of hospitalisation and iii) provision of a printout for carers including safety-netting advice.

Co-Primary outcomes: For 0-9 year olds - i) Dispensing data for amoxicillin and macrolide antibiotics and ii) hospital admission rate for RTI.

Results: We recruited 294 of the intended 310 practices (95%) representing 336,496 registered 0-9 year-olds (5% of all 0-9 year-old children). Included practices were slightly larger, had slightly lower baseline prescribing rates and were located in more deprived areas reflecting the national distribution. Engagement with CCGs and their understanding of their role in this research was variable. Engagement with CRNs and installation of the intervention was straight-forward although the impact of updates to practice IT systems and lack of familiarity required extended support in some practices. Data on the co-primary outcomes was almost 100%.

Conclusions: The infrastructure for trials at the practice level using routinely collected data for primary outcomes is viable in England and should be promoted for primary care research where appropriate.

Trial registration: ISRCTN11405239

For peer review only

Article Summary

Strengths and limitations of this study

- Utilising routinely collected data in primary care at the practice level removes the burden of individual patient recruitment and potential for selection bias.
- Using the NIHR Clinical Research Network across England and Clinical Commissioning Groups (CCGs) allowed recruitment of practices with a broader research experience.
- Embedding the intervention within practice systems utilises existing patient data.
- Engagement with third parties (such as the CCGs) to collect primary outcome data adds another layer of administrative burden.
- Data collected at the practice level are limited (e.g. absence of denominator data such as patients consulting for different conditions) so are only viable if suitable proxy markers can be found for the outcomes of interest.

For peer review only

Introduction

Conducting randomised controlled trials (RCTs) in primary care is essential for the development of a robust evidence base to improve the treatment, health and well-being of patients. However, it is a difficult environment in which to conduct research. In England, general practices are already divided into those who are willing to conduct research and those who are not which challenges the external validity of any successful trial when rolling out an intervention.[1] Effective recruitment strategies generally require practitioner involvement[2] but this is difficult in the time allowed for consultations and can lead to the exclusion of patients for whom recruitment might be more challenging and therefore increase the risk of selection bias.[3] The time pressure can be compounded if the intervention is not fully integrated into the practice computer systems; a stand-alone tool takes time to open and close, may not draw upon information already collected within the system and may be less amenable to modification on a wider scale. In Primary Care research there are also difficulties in collecting patient outcomes. This is largely dependent on physical access to patient notes and is both costly and time-consuming.

Conducting trials at the practice level removes the need for clinicians to recruit individual patients and opens up the possibility of utilising routinely collected data for patient groups at each practice. Nationally collected routine data by practice and patient age are available from Clinical Commissioning Groups (CCGs)[4] and reliable data related to activity or financial transactions, such as the dispensing of medications and hospitalisation rates can, be used for primary trial outcomes. The research infrastructure in England created by the National Institute for Health Research (NIHR) could make the wider recruitment of practices viable.[5] Clinical Research Networks (CRNs) support health and care organisations to be research active[6] and can help recruit practices at a national level, including those serving diverse socioeconomic populations, improving the generalisability of findings.[7] Over 90% of the 7526 GP practices in England use either the *Egton Medical Information Systems* (*EMIS*) (56%) or *SystmOne* (34%) and both have the facility to integrate intervention algorithms and utilise patient data already on the system.[8] Simpler designed studies, which place fewer demands on clinicians and practices compared with other studies, may also encourage research-naïve practices to take part in research.

Recruitment at the patient-level was found to be a challenge in our feasibility study with a significant differential in the health of the patients between arms, increased consultation times due to individual recruitment and using a stand-alone intervention and costly in terms of collecting individual patient notes. [9,10] The on-going main trial, which aims to reduce antibiotic prescribing among children presenting with an upper respiratory tract infection and cough (The CHIldren's COugh or CHICO trial) was re-designed at the practice level.[11] We report on our experience of a

For peer review only - http://bmjopen.bmj.com/site/about/guidelines.xhtml

simpler design; recruiting practices nationally utilising routinely collected data as the primary outcome, integrating the intervention within electronic health record systems and a light-touch approach to data collection. We also used a brief survey to find out how CRNs communicate with practices and semi-structured interviews of key individuals in CRNs and CCGs to look at the barriers and facilitators to this approach.

Methodology

Respiratory tract infections (RTIs) in children are common and present major resource implications for primary care.[12,13] Unnecessary use of antibiotics is associated with the development and proliferation of antimicrobial resistance. In 2016 our 5-year NIHR-funded 'TARGET' programme developed a prognostic algorithm to identify children with acute cough and RTI at very low risk of hospitalisation within 30 days and unlikely to need antibiotics.[14] The intervention included: i) explicit elicitation of parental concerns, ii) the results of the prognostic algorithm accompanied by prescribing guidance and iii) provision of support for a no-antibiotic strategy through a printout for carers including safety netting advice.[15]

The subsequent feasibility study, recruiting at the patient-level, showed prescribing reductions in both arms of the trial but also exposed the differential recruitment of significantly healthier children in the control arm. In the qualitative interviews, clinicians reported preferential recruitment of less unwell children as these were quicker to manage and therefore easier to recruit.[10]

To negate differential recruitment, and conserve resources, an 'efficient design' was proposed for the full trial. The main changes in design were:

i) Recruiting practices (with the help of CRNs and CCGs) rather than individual patients.
ii) Using routinely collected data from CCGs and from national reporting systems for the primary outcome rather than directly from the practices.
iii) Integrating the intervention within electronic medical records (with a triggered pop-up) rather than a stand-alone web-based tool.
iv) A light-touch approach to collecting secondary data using practice champions (eliciting the help of a practice manager or someone familiar with practice systems) rather than accessing patient notes.

The CHICO RCT is an efficient, pragmatic open label, two-arm (intervention vs. usual care) trial with an embedded qualitative study, with randomisation at the practice level, using routine antibiotic dispensing and hospitalisation data to assess effectiveness. The study population is children aged 0-9 years presenting with acute cough and RTI. Oral suspensions are more often given to this age group.

For peer review only

The setting is consultations in primary care practices with prescribing clinicians in diverse regions across England. Recruitment is at the practice level, so consent is not required for individual participants. Recruitment of practices is via CCGs and by using the Clinical Research Network (CRN) who support patients, the public and health and care organisations across England to participate in high-quality research. Research ethics approval was given by London-Camden and Kings Cross Research Ethics Committee (ref:18/LO/0345). This research is funded by the National Institute for Health Research (NIHR) Health Technology Assessment (HTA) programme (funder ref: 16/31/98).

Feedback on the roles of CRNs and CCGs in recruiting practices were obtained from a short questionnaire sent to all CRNs and semi-structured interviews with a convenience sample of key individuals in CRNs and CCGs. The questionnaire focussed on how CRNs communicate with practices and the subsequent interviews explored these points and individual opinions of conducting efficient design trials in primary care in greater depth. The interviews were conducted in September 2021. Questionnaire responses were summarized in a table and pertinent comments highlighted. Interviews were audio-recorded, transcribed verbatim and analysed using a framework approach.[16] Transcripts were all read for familiarisation with the data and an initial sample of transcripts using both deductive (key issues in the interview topic guide), and inductive (derived from the data) codes. The initial codes were discussed with the wider team and a thematic framework was developed and used to code the whole of the dataset. Findings were then summarised in table format and used to inform wider understanding of the facilitators and barriers to conducting CHICO.

Patient and Public Involvement

This intervention has been developed collaboratively with our parent advisory group (PAG) and clinical advisory group (CAG) throughout the 'TARGET' programme. Their comments and suggestions about the format of the intervention and parent/carer materials have informed the intervention and the design of the earlier feasibility study and the design of the main trial.

Results

i) Recruiting practices

The sample size calculation indicated we needed 310 practices (155 intervention, 155 usual care). Between October 2018 and October 2020, we recruited 294 practices (94.8%) representing 336,496 registered patients aged 0-9 years old (5.0% of all 0-9 year-old children in England).[17] Practices were recruited using all 15 CRNs in England and 47 of the 211 CCGs covering the English regions in 2019 (Figure 1).[18] Table 1 gives an indication of the generalisability of the results. Over half of the practices (59%) were larger than the average list size in England, around a third (32%) had higher prescribing

For peer review only

rates than the national average and over a quarter of the practices (26%) were in the most deprived socio-economic quintile reflecting the national distribution of more practices being located in urban areas.

Table 1. Practice baseline characteristics

	n	CHICO practices (n=294) n (%) or *Median (IQR)*
Total number of 0-9 year olds listed[a]		339,496
Practice characteristics, using routine data sources		
Above the average list size in England[b]	294	173 (59%)
Median list size of 0-9 year olds (IQR)[a]	294	*984 (678, 1405)*
Above the median prescribing rate in England[c]	294	93 (32%)
Median prescribing rate (IQR)[d]	294	*17.9 (14.3, 24.6)*
Patient characteristics, using a practice questionnaire [e]		
Median # of General Practitioners	290	*6.0 (4.0, 9.0)*
Median # of salaried nurses	238	*2.0 (1.0, 4.0)*
Median # of sessional nurses	133	*0.0 (0.0, 1.0)*
Median # pharmacist independent prescribers	190	*1.0 (0.0, 1.0)*
Median # of Locums in previous 12 months	203	*3.0 (2.0, 6.0)*
Median distance to the nearest childrens A&E (miles)	287	*4.5 (2.2, 10.0)*
IMD[f] quintile based on practice postcode		
1 (most deprived)		77 (26%)
2		60 (20%)
3	294	60 (20%)
4		54 (18%)
5 (least deprived)		43 (15%)
Income Deprivation Affecting Children Index Score[g]		
Median proportion (IQR)	294	*15% (8%, 26%)*

[a] Based on the mean practice list size of 0-9 year olds over the 12-months prior to randomisation, [b] The median practice list size of 0-9 year olds in England in October 2019, the mid-point of recruitment, was 852 patients[14] , [c] Based on amoxicillin and macrolide use in England CCGs (median=22) for all ages[19]. [d] Based on the total number of amoxicillin/macrolide items dispensed over the 12-months prior to randomisation, divided by the practice list size, [e] As reported by the practice champion, [f] index of multiple deprivation, [g] The Income Deprivation Affecting Children Index (IDACI) measures the proportion of all children aged 0-15 living in income deprived families. It is a subset of the Income Deprivation Domain which measures the proportion of the population in an area experiencing deprivation relating to low income. The definition of low income used includes both those that are out-of-work, and those that are in work but who have low earnings (and satisfy the respective means tests).

Assuming most families live close to their GP practice, using Income Deprivation Affecting Children Index (IDACI), we estimate 15% of children included in the trial lived in a deprived neighbourhood (defined as those people that are out-of-work, and those that are in work but who have low earnings). Typically, the number of registered 0-9 year olds was just under 1000 per practice, staffed with a median of 6 general practitioners, 2 salaried nurses, 1 pharmacist and 3 locums over one year. The practice list size of 0-9 year old children ranged from 149 to 6969 with 64 practices (22%) having more than 1500 children registered (Figure 2a). The number of amoxicillin or macrolides dispensed, over the 12-month baseline period prior to randomisation ranged from <5 to >55 per 100 patients (Figure 2b) with a median of 18 items (Table 1). Recruitment was planned over a 12-month period but took 24 months to complete. This in part was due to first having to obtain agreement from CCGs (providing the co-primary outcomes) to participate in the study and delays due to the COVID-19 pandemic.

For peer review only

Facilitators

The 15 CRNs were helpful with the recruitment of practices (Table 2); presenting our study at CRN meetings and using the national CRN lead for support helped facilitate a co-ordinated national approach.

Table 2. Semi-structured interviews with members of CRNS and CCG

Question	Response
What are the facilitators to practice recruitment?	*"Communicating with the practices. I think in an academic centre, if you're writing to practices, they don't really know you, do they? Whereas actually, if we put it in our local bulletins and keep encouraging practices to think about it, then it's probably a better way of increasing uptake."* [P4 CCG]<br><br>*"There's been a nice friendship [with study team] and trying to create the right words to share with practices."* [P1 CCG]<br><br>*"Just keep on plugging. I think quite often they're either going through a really busy time, I've contacted the wrong person or there's not enough information there for them to make a snap decision. The more information I've got and the simpler I make it, then the more likely they are to say yes or no."* [P3 CRN]
What are the barriers to practice recruitment?	*"To be honest it was a little bit more difficult to get the paperwork signed by the CCG as well within the practices, 'cause a lot of the practices we generally have a named contact, someone who already knows us and we've got relationships with…so it was a lot easier to get engagement than it was from CCGs."* [P2 CRN]<br><br>*"I try to avoid the practice managers making the decisions if I can because they are good gatekeepers. Well our practice is very busy, that GP won't be interested when actually sometimes they quite often are."* [P3 CRN]
Tell us about Research active and non-active (naïve) practices?	*"It's usually the research active sites that get back to us but sometimes others do respond and want to take part in studies and we've got this ongoing engagement programme with all the practices in ((city)) to try and get more of them on board with research even if it's just doing simple stuff."* [P5 CRN]<br><br>*"I would say that about a third of the practices were what I would call research naïve or inexperienced, green as in they hadn't really had a sort of established relationship with us in the past."* [P2 CRN]
Tell us about the role of CCGs in Research?	*"I mean in reality they [CCG] don't play a major part in – and never actually have a major role in research. It's not a core business of a CCG like it would be in a provider. So we don't have a research department, which is probably why the CHICO trial ended up at my door because it was about prescribing."* [P4 CCG]
Tell us about efficient design trials?	*"Yeah, go for it because once you've got the practice on board, it's almost like they don't have to do as much either, I would definitely encourage practices to take on this type of research, definitely, as long as you've got everybody on board and it all works through then yeah, there's no reason why not."* [P3 CRN]
	"It seems quite suited to primary care because I think primary care's biggest issue is time. So, if they've got a patient in front of them, the chance of them actually getting that patient to consent is probably quite low because it's just they – that's an extra minute on a ten-minute appointment isn't it really?" [P4 CCG]

Feedback from 11 of the 15 CRNs indicated that some contact practices that opt-in to conduct research whilst others contact all the practices in their area; around half the practices had joined

the Research Sites Initiative scheme (NIHR funded scheme to enable research delivery) and were often the first to be contacted. Recruitment via CCGs had a wider reach of practices than via CRNs, although CCG participation in recruitment was more variable depending on capacity. Using quarterly study newsletters for practices, CRNs and CCGs with league tables monitoring levels of recruitment improved responses.

Barriers

Limiting the contact to practices that want to opt-in to research via some of the CRNs misses the opportunity of letting research-naïve practices know about light-touch efficient design studies. The level of engagement from CRNs varied slightly but much more between CCGs. Some CCGs were averse to getting involved in research or cited lack of capacity as a reason to be excluded from the research (Table 2). It was difficult to know which individuals to contact and how to do so (some CCGs did not appear to be public-facing), response times were often slow, their role in research was often misunderstood, staff changes hindered communication and a number of CCGs merged during the study period. At the start of recruitment, October 2018, there were 211 CCGs across England. By the end of follow up, September 2021, there were only 106 CCGs. While this did reduce the number of CCG contacts required to obtain the data, it sometimes resulted in change of staff who were not familiar with the trial or its requirements. Of the 294 practices we recruited, we are aware of at least 22 practices (7%) who merged during their baseline/follow up annual data capture. We excluded practices who anticipated a merger with another practice but had no control over this once the practice was randomised, especially in the rare instances when the merging practices were randomised to different arms of the trial. The length of time from expression of interest from practices to randomisation was longer than expected due to the delayed site agreements returned from the practices.

<u>ii) Using routinely collected data for the primary outcomes</u>

The co-primary outcomes in the trial were i) practice dispensed prescription data for amoxicillin and macrolide antibiotics for children 0-9 years and ii) hospital admission rate for RTI among 0-9 year olds. The antibiotic dispensing rates were retrieved from the *NHSBSA ePACT2* reporting system for all practices in the trial and practice list size data by 5 year epoch was retrieved from *NHS Digital* and combined to create a dispensed prescription rate for amoxicillin and for macrolide antibiotics.[20] The hospital admission rates are routinely collected by all English CCGs. The ascertainment of these data was 99.7% (293/294) for antibiotic dispensing and hospitalisation rates. Data for one practice was lost due to a merger.

For peer review only

Facilitators

Using aggregated data avoids the need for the consulting physician to solicit individual consent, reduces or eliminates the risk of selection bias and removes the task of accessing individual patient notes. Routine data are often collected monthly so data completeness and quality can be monitored throughout the collection period, which was particularly important in this trial to scrutinise and report any sudden changes in hospital admission rates to the Data Monitoring Committee. The routinely collected data can be imported to data management software, thus there is less likelihood of data entry errors and missing data are rare. The cost of research staff and time taken to collect the primary outcome data was much reduced compared to a traditional trial. In total 11/294 practices asked to be withdrawn from the study (3.7%) and 14/294 (4.8%) were lost to follow-up from the study; the main reasons cited being lack of capacity and prioritising the COVID-19 pandemic. A further advantage of collecting routine data is that withdrawal of a practice does not necessarily mean a loss of the primary outcome data for that practice (if the data are already in the public domain).

Barriers

A potential problem with aggregate data collected from a third party is where data are suppressed, owing to a low number of events, although in this study data were collected over a 12-month period largely avoiding this problem. Some liaison was needed between the trial team and the CCGs to know exactly what data were available and in what format this could be presented. Dispensed prescription data, practice list size data and hospital admission data are reliable as they feed into financial transactions, however data reporting emergency department [ED] attendance were less so due to a limited coding set. Many ED attendances do not have a diagnosis coded, therefore the number of attendances attributable to RTI is likely to be inaccurate.

iii) Integrating the intervention within the electronic medical record system

The trial only included practices using the *EMIS* system. The practice champion was given written instructions on how to install the intervention within *EMIS*. Self-directed training materials were provided for all clinicians using the intervention. The algorithm consists of seven predictors of future hospitalisation, two of which were already in the practice systems (child age and history of asthma) and thus pulled into the algorithm automatically from patient records, and five which were entered during consultation (illness duration, raised temperature, vomiting in last 24 hours, presence of wheeze and presence of intercostal or subcostal recession).[21] Carer concerns were elicited during the consultation and formed part of a personalised leaflet generated from the system containing an easy to read 'Caring for Children with Coughs' graphic and safety-netting advice about when to seek

For peer review only

medical care or advice.[16] Intervention use was monitored using searches on the *EMIS* system, run by the practice champion and shared with the research team.

Facilitators

Installation of the intervention was relatively straight-forward for *EMIS*. Practice systems allow for user-friendly manipulation so interventions can be integrated and interface with system data already collected, thus negating any additional clinical workload. Screen pop-ups can also be used to notify clinicians of eligible patients to the study.

Barriers

Familiarity with the *EMIS* system varied between practices thus the level of support required from the research team to help install the intervention and download usage also varied. Provision to help install or use third-party algorithms is not offered by system providers. EMIS upgrades to the system during the trial meant that rewriting installation instructions, resources and testing had to be carried out and fed back to the practices. For instance, the READ codes used to identify clinical terms within consultations were upgraded by EMIS in the early months of 2020 which meant the algorithm had to be amended so the intervention would function correctly. We found that our funded provision of IT support throughout the study was crucial to the smooth running of the integrated intervention. Although pop-ups can be used, some clinicians found them irritating and switched them off whilst other practices had so many pop-ups (especially during the COVID-19 pandemic) that the CHICO pop-up was often obscured.

<u>iv) A light-touch approach to data collection</u>

Clinicians were not required to provide any data about individual participants (apart from reporting serious adverse events), those in the intervention group were asked to familiarise themselves with the tool and use it, whilst those in the control arm were asked to provide usual care. Data collection from the practices directly was limited to short baseline and follow-up questionnaires. Data on intervention usage were downloaded from *EMIS* and the co-primary outcome downloads from ePACT2 and the relevant CCGs.

Facilitators

The light touch approach reduced the time needed during consultation to record information, the trawling of patient notes and provided a more objective data resource downloaded from the system rather than from individual input. Practice champions, familiar with practice systems, played an important role in obtaining the required data. Baseline questionnaires were received from 294/294 practices (100%), follow-up questionnaires were received from 265/294 practices (90%) whilst

For peer review only - http://bmjopen.bmj.com/site/about/guidelines.xhtml

intervention usage was collected from 116/144 of the intervention practices (81%), indicating the data burden was not too onerous.

Barriers

Conversely, this approach reduced the level of interpretation that can be gleaned from the data. Removing patient level recruitment results in a loss of denominator data, in our case not knowing how many individual children consulted for RTI and cough. As a proxy we used the number of children registered at each practice. Given the diversity of the practices included in the trial we are assuming that those children taking part in the trial were no different from children in the general population, but we have no way of testing this assumption. We also lost detail that may be taken from the consultation of whether antibiotics were prescribed immediately or delayed relying instead on the number of antibiotics being dispensed. The lack of contact with usual care practices (apart from reminders to provide SAE reports) runs the risk of practices wanting to withdraw from the study, especially with changes of staff. Consideration also needs to be given to the ethical implications of not seeking consent from individual patients.

Discussion

The National Institute for Health Research (NIHR) is keen to see the design, development and delivery of more efficient, faster, innovative studies which provide robust evidence to inform clinical practice and policy.[22] The CHICO study demonstrates that an efficiently designed practice-level large trial in primary care using routinely collected data is feasible and potentially good value for money. The average cost of an HTA RCT was £1.25 million in 2019/20,[23] whereas the cost of the CHICO RCT, which included over 300,000 children (5% of the entire national 0-9 year-old population) and 4% of all GP practices in England, was below £1 million. Utilising routinely collected data as the primary outcome reduces problems with missing data whilst removing the burden of patient recruitment and focusing the clinician's time on using the intervention reflects real life practice. These findings are pertinent to the health care system in England but might lend themselves to similar primary care networks in other countries.

Integrating the intervention within the practice system both exploits the data already available and adds to the patient's record avoiding duplicating of effort and saves time. Primary care practices are often very busy and the average length of face-to-face consultation in the UK is around 10 minutes; less than half the time given to patients in, for example, Sweden and the US.[24] Growing demands on

For peer review only

primary care services have also led to policymakers promoting telephone and video consultations, even before the COVID-19 pandemic, and these sometimes don't lend themselves to enlisting patients in research.[25] Reducing the research burden for participants and clinicians is always desirable, but particularly so given the increasing time constraints in primary care. This light-touch approach may also be more appealing for practices who maybe research naïve; investing in different recruitment strategies[26] using existing networks could potentially yield a more representative sample than previous trials in primary care from which to generalise any findings. The design of the CHICO trial retrospectively scored highly in each domain of the PRECIS-2 ((PRagmatic Explanatory Continuum Indicator Summary) tool[27] suggesting it is a pragmatic randomised trial focusing on delivery in the "real world" rather than providing the best chance to demonstrate a beneficial effect in an idealised setting.

Efficient design studies which utilise routine data and recruit at practice level face their own challenges. First, this approach would not be suitable if individual patient consent is required or for trials using a patient reported outcome as the primary outcome, as these are not widely collected in routine care. Furthermore, the number of CCGs in England almost halved during the study period due to organisational restructuring which made it difficult to administer the trial. From March 2022 Integrated Care Systems will replace CCGs and this will provide new bureaucracy for researchers to develop relationships with. There is a lack of uniformity when approaching these commissioning groups and their role in research needs clarifying.[28] Using a convenience sample the qualitative interviews have limited insight but suggest research needs to be higher on the agenda. CRNs are more research-focused and can help with recruitment although adoption of the study needs to be made more explicit and a consistent national approach to include research naïve practices needs to be adopted within this network.

If practices are being used as the unit of analysis, then the commitment to research they have signed up for needs to be strengthened. Around 2.5% practices close or merge each year in England whilst some new practices open and recognition of the current research portfolio needs to be part of this process.[29] We were also surprised by the wide variability in practice list size, 8% of practices in the study having 3 sites or more. This has implications for future trials in terms of factoring in variable list sizes for sample size calculations and checking that multiple sites use the same electronic record systems. A light touch approach is only viable if suitable primary outcomes can be identified but makes fidelity more difficult to measure. Losing denominator data such as patients consulting for different conditions will depend on the hypothesis being tested and needs to strike a balance

For peer review only

between accuracy of what you are trying to measure and whether a proxy marker will deliver the population impact of the intervention. Using the patient list size of 0-9 year olds as a proxy denominator instead of those consulting for RTI accounts for the variability between the size of different practices but not necessarily the disease burden.

If one of the main intentions of primary care research is to provide the clinician with better tools, then we need to work more closely with those who supply the toolbox (IT system providers) and the third parties who provide the data within which practices work. The infrastructure to conduct efficiently designed trials that do not require patient-reported outcomes in England is potentially viable but does require more investment in time and effort to make recruitment of practices and data collection more accessible to researchers.

For peer review only

Authors Contributions

AH, PB, PL, NF and JI were responsible for developing the research questions. PB, AH, JI, PL, CCab, CClem, EB, MG, JH, SC, AL and NF and are responsible for the study design and collection of data. PS, JT and SB are responsible for study management and coordination. GY, PD and CClem are responsible for the analysis of the data. PB drafted the paper. All authors read, commented on and approved the final manuscript.

Acknowledgements

This study was designed and delivered in collaboration with the Bristol Randomised Trials Collaboration (BRTC), part of the Bristol Trials Centre, is in receipt of National Institute for Health Research CTU support funding. The University of Bristol is acting as the sponsor for this trial and the trial is hosted by the NHS Bristol, North Somerset and South Gloucestershire Clinical Commissioning Group (CCG). The authors would like to thank all General practices, CCGs and CRNs for their involvement in CHICO. The authors would also like to thank the members of the TSC and DMC.

Funding

This research is funded by the National Institute for Health Research (NIHR) Health Technology Assessment (HTA) programme (funder ref: 16/31/98). The views expressed are those of the authors and not necessarily those of the NIHR or the Department of Health and Social Care.

Competing interest

None declared

Data Sharing

The data are obtained from a third party (Clinical Commissioning Groups and Public Health England) and are not publicly available

Ethics Approval

Research ethics approval was given by London-Camden and Kings Cross Research Ethics Committee (ref:18/LO/0345).

For peer review only

References

1) Allcott H. Site Selection Bias in Program Evaluation. *The Quarterly Journal of Economics* August 2015;130 (3): 1117–1165.
2) Ngune I, Jiwa M, Dadich A, Lotriet J, Sriram D. Effective recruitment strategies in primary care research: a systematic review. *Qual Prim Care* 2012;20(2):115-23
3) Foster JM, Sawyer SM, Smith L, Reddel HK, Usherwood T. Barriers and facilitators to patient recruitment to a cluster randomized controlled trial in primary care: lessons for future trials. *BMC Med Res Methodol* 2015 Mar 12;15:18
4) Clinical Commissioning Groups https://www.england.nhs.uk/ccgs/
5) National Institute for Health Research https://www.nihr.ac.uk/researchers/collaborations-services-and-support-for-your-research/
6) Clinical Research Network https://www.nihr.ac.uk/explore-nihr/support/clinical-research-network.htm
7) Bower P, Grigoroglou C, Anselmi L, Kontopantelis E, Sutton M, Ashworth M, Evans P, Lock S, Smye S, Abel K. Is health research undertaken where the burden of disease is greatest? Observational study of geographical inequalities in recruitment to research in England 2013-2018. *BMC Med* 2020 May 18;18(1):133.
8) Kontopantelis E, Stevens RJ, Helms PJ, Edwards D, Doran T, Ashcroft DM. Spatial Distribution of Clinical Computer Systems in Primary Care in England in 2016 and Implications for Primary Care Electronic Medical Record Databases: A Cross-Sectional Population Study. *BMJ Open* 2018 Feb 28;8(2):e020738
9) Turnbull SL, Redmond NM, Lucas P, Cabral C, Ingram J, Hollinghurst S, Hay AD, Peters TJ, Horwood J, Little P, Francis N, Blair PS. The CHICO (Children's Cough) Trial protocol: a feasibility randomised controlled trial investigating the clinical and cost-effectiveness of a complex intervention to improve the management of children presenting to primary care with acute respiratory tract infection. *BMJ Open* 2015 Sep 15;5(9):e008615.
10) Blair PS, Turnbull S, Ingram J, Redmond N, Lucas PJ, Cabral C, Hollinghurst S, Dixon P, Peters T, Horwood J, Little P, Francis NA, Gilbertson A, Jameson C, Hay AD. Feasibility cluster randomised controlled trial of a within-consultation intervention to reduce antibiotic prescribing for children presenting to primary care with acute respiratory tract infection and cough. *BMJ Open* 2017 May 9;7(5): e014506.
11) Seume P, Bevan S, Young G, Ingram J, Clement C, Cabral C, Lucas PJ, Beech E, Taylor J, Horwood J, Dixon P, Gulliford M, Francis NA, Creavin S, Lane AJ, Hay A, Blair PS. Protocol for an ‘efficient design’ cluster randomised controlled trial to evaluate a complex intervention to improve antibiotic prescribing for CHIldren presenting to primary care with acute COugh and respiratory tract infection: The CHICO study. *BMJ Open.* 2021 Mar 29;11(3): e041769.
12) Smith DRM, Dolk FCK, Pouwels KB, Christie M, Robotham JV, Smieszek T. Defining the appropriateness and inappropriateness of antibiotic prescribing in primary care. *J Antimicrob Chemother* 2018 Feb 1;73(suppl_2): ii11-ii18.
13) Vodicka TA, Thompson M, Lucas P, Heneghan C, Blair PS, Buckley DI, Redmond N and Hay AD. Reducing antibiotic prescribing for children with respiratory tract infections in primary care: a systematic review. *Br J Gen Pract* 2013 Jul;63(612): e445-54.
14) Hay AD, Redmond NM, Turnbull S, Christensen H, Thornton H, Little P, Thompson M, Delaney B, Lovering AM, Muir P, Leeming JP, Vipond B, Stuart B, Peters TJ, Blair PS. Development and internal validation of a clinical rule to improve antibiotic use in children

For peer review only

presenting to primary care with acute respiratory tract infection and cough: a prognostic cohort study. *Lancet Respir Med* 2016 Nov;4(11):902-910.
15) Lucas PJ, Ingram J, Redmond NM, Cabral C, Turnbull SL, Hay AD. Development of an intervention to reduce antibiotic use for childhood coughs in UK primary care using critical synthesis of multi-method research. *BMC Med Res Methodol*. 2017 Dec 28;17(1):175.
16) Ritchie J, Spencer L. Qualitative data analysis for applied policy research. In: A. Bryman and R. G. Burgess, ed. Analyzing qualitative data1994:173-94.
17) Population estimate of 0-9 year olds in England using October 2019 (mid-point of recruitment) https://digital.nhs.uk/data-and-information/publications/statistical/patients-registered-at-a-gp-practice/october-2019
18) CCG annual assessment 2019/20 © NHS England and NHS Improvement 2020 Publication approval reference: PAR112 https://www.england.nhs.uk/wp-content/uploads/2020/11/ccg-annual-assessment-report-19-20.pdf
19) Estimate of antibiotic items prescribed per CCG (October 2017 to September 2018) https://openprescribing.net/analyse/#org=CCG&orgIds=15E,15C,D2P2L,14Y,06H,04Y,27D,15N,01A,05D,97R,D4U1Y,11M,D9Y0V,06N,91Q,11N,15F,99A,14L,01K,13T,06T,05G,W2U3Z,52R,10Q,01G,05Q,11X,M2L0M,05W,72Q,36L,05V,00P,92A,03Q&numIds=0501013B0,5.1.5&denom=total_list_size&selectedTab=summary
20) ePACT2 prescribing data held by NHS Prescription Services. https://www.nhsbsa.nhs.uk/epact2
21) Hay AD, Redmond NM, Turnbull S, Christensen H, Thornton H, Little P, Thompson M, Delaney B, Lovering AM, Muir P, Leeming JP, Vipond B, Stuart B, Peters TJ, Blair PS. Development and internal validation of a clinical rule to improve antibiotic use in children presenting to primary care with acute respiratory tract infection and cough: a prognostic cohort study. *Lancet Respir Med*. 2016 Nov;4(11):902-910.
22) NIHR: Annual Efficient Studies funding calls for CTU projects. https://www.nihr.ac.uk/documents/ad-hoc-funding-calls-for-ctu-projects/20141
23) NIHR Annual Report 2019/20. Department of Health & Social Care (page 5) https://www.nihr.ac.uk/documents/about-us/our-contribution-to-research/research-performance/NIHR_Annual_Report_19_20.pdf
24) Irving G, Neves AL, Dambha-Miller H, Oishi A, Tagashira H, Verho A, Holden J. International variations in primary care physician consultation time: a systematic review of 67 countries. *BMJ Open* 2017 Nov 8;7(10):e017902
25) Hammersley V, Donaghy E, Parker R, McNeilly H, Atherton H, Bikker A, Campbell J, McKinstry B. Comparing the content and quality of video, telephone, and face-to-face consultations: a non-randomised, quasi-experimental, exploratory study in UK primary care. *Br J Gen Pract* 2019 Aug 29;69(686):e595-e604
26) Ellis SD, Bertoni AG, Bonds DE, Randall Clinch C, Balasubramanyam A, Blackwell C, Chen H, Lischke M, Goff Jr DC. Value of recruitment strategies used in a primary care practice-based trial. *Contemp Clin Trials* 2007 May;28(3):258-67.
27) Loudon K, Treweek S, Sullivan F, Donnan P, Thorpe KE, Zwarenstein M. The PRECIS-2 tool: designing trials that are fit for purpose. *BMJ*. 2015 May 8;350:h2147
28) Checkland K, McDermott I, Coleman A, Perkins N. Complexity in the new NHS: longitudinal case studies of CCGs in England. *BMJ Open* 2016 Jan 7;6(1):e010199
29) Practices closing or merging

For peer review only

https://digital.nhs.uk/data-and-information/find-data-and-publications/supplementary-information/2018-supplementary-information-files/number-of-gp-practices-closed-or-merged-in-england-between-1-july-2016-and-31-december-2017-by-nhs-geographies

For peer review only

Figure 1. The CCGs taking part in the CHICO study (bold), shaded according to the number of items of amoxicillin and macrolides, per 1000 list size at the mid point of recruitment (Oct '19).

Figure 2a. The distribution of practice list sizes*, of 0-9 year olds, for those practices taking part in the CHICO study

Figure 2b. The distribution of practice dispensing rates*at baseline, for 0-9 year olds, for those practices taking part in the CHICO study

For peer review only

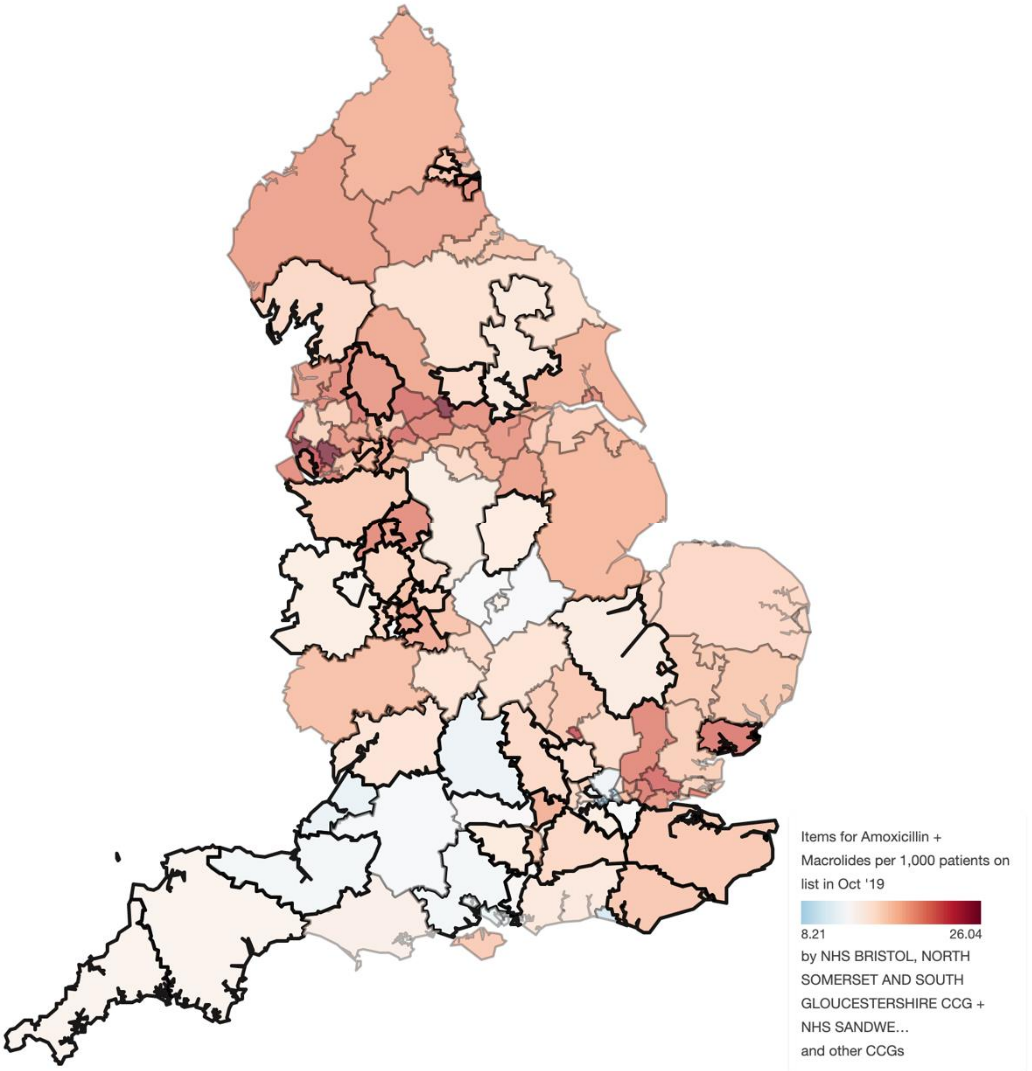

Source: OpenPrescribing.net, EBM DataLab, University of Oxford, 2017

For up to date data please refer to:
[https://openprescribing.net/analyse/#org=CCG&orgIds=15E,15C,D2P2L,14Y,06H,04Y,27D,15N,01A,05D,97R,D4U1Y,11M,D9Y0V,06N,91Q,11N,15F,99A,14L,01K,13T,06T,05G,W2U3Z,52R,10Q,01G,05Q,11X,M2L0M,05W,72Q,36L,05V,00P,92A,03Q&numIds=0501013B0,5.1.5&denom=total_list_size&selectedTab=map]

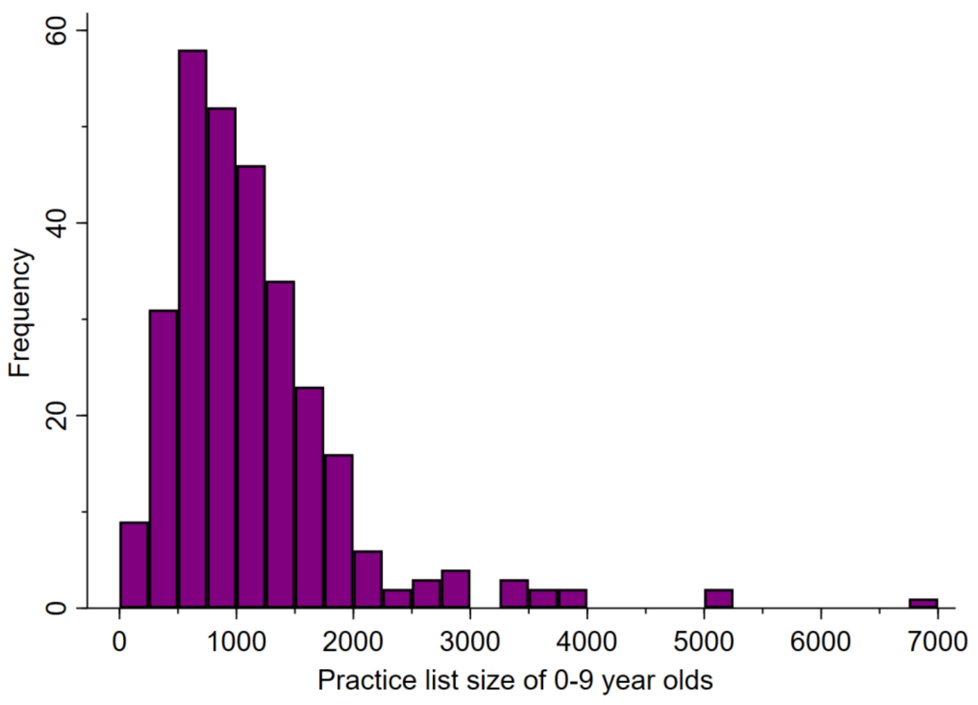

*Defined for each practice as the mean list size of 0-9 year olds, over the 12 months prior to randomisation (source: https://digital.nhs.uk/data-and-information/publications/statistical/patients-registered-at-a-gp-practice)

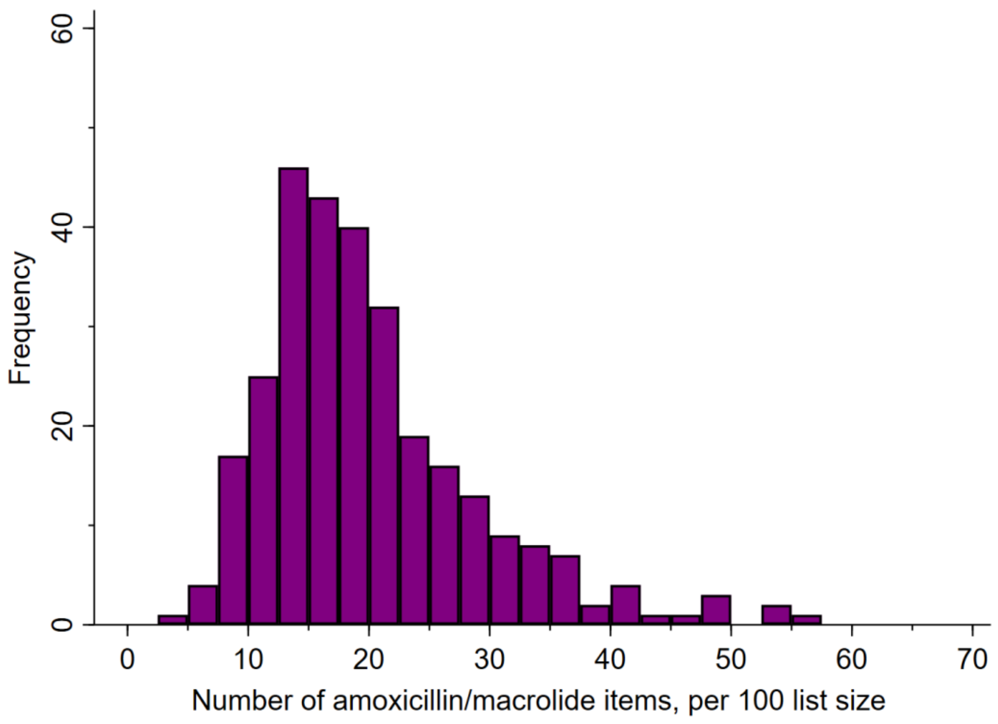

*Defined for each practice as the number of amoxicillin or macrolides dispensed, over the 12-month baseline period prior to randomisation, divided by the practice list size. This may include multiple items per child.

CONSORT 2010 checklist of information to include when reporting a randomised trial*

Section/Topic	Item No	Checklist item	Reported on page No
**Title and abstract**			
	1a	Identification as a randomised trial in the title	1
	1b	Structured summary of trial design, methods, results, and conclusions (for specific guidance see CONSORT for abstracts)	2
**Introduction**			
Background and objectives	2a	Scientific background and explanation of rationale	4-5
	2b	Specific objectives or hypotheses	4-5
**Methods**			
Trial design	3a	Description of trial design (such as parallel, factorial) including allocation ratio	5
	3b	Important changes to methods after trial commencement (such as eligibility criteria), with reasons	N/A^
Participants	4a	Eligibility criteria for participants	N/A^
	4b	Settings and locations where the data were collected	5
Interventions	5	The interventions for each group with sufficient details to allow replication, including how and when they were actually administered	5
Outcomes	6a	Completely defined pre-specified primary and secondary outcome measures, including how and when they were assessed	N/A^
	6b	Any changes to trial outcomes after the trial commenced, with reasons	N/A^
Sample size	7a	How sample size was determined	N/A^
	7b	When applicable, explanation of any interim analyses and stopping guidelines	N/A^
Randomisation:			
Sequence generation	8a	Method used to generate the random allocation sequence	N/A^
	8b	Type of randomisation; details of any restriction (such as blocking and block size)	N/A^
Allocation concealment mechanism	9	Mechanism used to implement the random allocation sequence (such as sequentially numbered containers), describing any steps taken to conceal the sequence until interventions were assigned	N/A^
Implementation	10	Who generated the random allocation sequence, who enrolled participants, and who assigned participants to interventions	N/A^
Blinding	11a	If done, who was blinded after assignment to interventions (for example, participants, care providers, those	N/A^

For peer review only - http://bmjopen.bmj.com/site/about/guidelines.xhtml

		assessing outcomes) and how	
	11b	If relevant, description of the similarity of interventions	N/A^
Statistical methods	12a	Statistical methods used to compare groups for primary and secondary outcomes	N/A^
	12b	Methods for additional analyses, such as subgroup analyses and adjusted analyses	N/A^
**Results**			
Participant flow (a diagram is strongly recommended)	13a	For each group, the numbers of participants who were randomly assigned, received intended treatment, and were analysed for the primary outcome	6
	13b	For each group, losses and exclusions after randomisation, together with reasons	9-10
Recruitment	14a	Dates defining the periods of recruitment and follow-up	6
	14b	Why the trial ended or was stopped	N/A
Baseline data	15	A table showing baseline demographic and clinical characteristics ~~for each group~~	7
Numbers analysed	16	For each group, number of participants (denominator) included in each analysis and whether the analysis was by original assigned groups	N/A^
Outcomes and estimation	17a	For each primary and secondary outcome, results for each group, and the estimated effect size and its precision (such as 95% confidence interval)	N/A^
	17b	For binary outcomes, presentation of both absolute and relative effect sizes is recommended	N/A^
Ancillary analyses	18	Results of any other analyses performed, including subgroup analyses and adjusted analyses, distinguishing pre-specified from exploratory	N/A^
Harms	19	All important harms or unintended effects in each group (for specific guidance see CONSORT for harms)	N/A^
**Discussion**			
Limitations	20	Trial limitations, addressing sources of potential bias, imprecision, and, if relevant, multiplicity of analyses	9-14 (barriers)
Generalisability	21	Generalisability (external validity, applicability) of the trial findings	N/A^
Interpretation	22	Interpretation consistent with results, balancing benefits and harms, and considering other relevant evidence	N/A^
**Other information**			
Registration	23	Registration number and name of trial registry	2
Protocol	24	Where the full trial protocol can be accessed, if available	16
Funding	25	Sources of funding and other support (such as supply of drugs), role of funders	6

^Not applicable for this manuscript, where the pros/cons of the trial design are presented. For more information on these items, please refer to the protocol: 10.1136/bmjopen-2020-041769

*We strongly recommend reading this statement in conjunction with the CONSORT 2010 Explanation and Elaboration for important clarifications on all the items. If relevant, we also recommend reading CONSORT extensions for cluster randomised trials, non-inferiority and equivalence trials, non-pharmacological treatments, herbal interventions, and pragmatic trials. Additional extensions are forthcoming: for those and for up to date references relevant to this checklist, see www.consort-statement.org.

For peer review only - http://bmjopen.bmj.com/site/about/guidelines.xhtml
For peer review only
